# Widespread white matter microstructural abnormalities in bipolar disorder: evidence from mega- and meta-analyses across 3033 individuals

**DOI:** 10.1038/s41386-019-0485-6

**Published:** 2019-08-21

**Authors:** Pauline Favre, Melissa Pauling, Jacques Stout, Franz Hozer, Samuel Sarrazin, Christoph Abé, Martin Alda, Clara Alloza, Silvia Alonso-Lana, Ole A. Andreassen, Bernhard T. Baune, Francesco Benedetti, Geraldo F. Busatto, Erick J. Canales-Rodríguez, Xavier Caseras, Tiffany Moukbel Chaim-Avancini, Christopher R. K. Ching, Udo Dannlowski, Michael Deppe, Lisa T. Eyler, Mar Fatjo-Vilas, Sonya F. Foley, Dominik Grotegerd, Tomas Hajek, Unn K. Haukvik, Fleur M. Howells, Neda Jahanshad, Harald Kugel, Trine V. Lagerberg, Stephen M. Lawrie, Julia O. Linke, Andrew McIntosh, Elisa M. T. Melloni, Philip B. Mitchell, Mircea Polosan, Edith Pomarol-Clotet, Jonathan Repple, Gloria Roberts, Annerine Roos, Pedro G. P. Rosa, Raymond Salvador, Salvador Sarró, Peter R. Schofield, Mauricio H. Serpa, Kang Sim, Dan J. Stein, Jess E. Sussmann, Henk S. Temmingh, Paul M. Thompson, Norma Verdolini, Eduard Vieta, Michele Wessa, Heather C. Whalley, Marcus V. Zanetti, Marion Leboyer, Jean-François Mangin, Chantal Henry, Edouard Duchesnay, Josselin Houenou, for the ENIGMA Bipolar Disorder Working Group

**Affiliations:** 1 Neurospin, CEA, Université Paris-Saclay, Gif-sur-Yvette, France; 2 INSERM Unit U955, Team 15, “Translational Psychiatry”, Créteil, France; 3 Assistance Publique-Hôpitaux de Paris (AP-HP), Corentin-Celton Hospital, Department of Psychiatry, Issy-les-Moulineaux, France; 4 Paris Descartes University, PRES Sorbonne Paris Cité, Paris, France; 5 Pôle de psychiatrie, DHU PePSY, Hôpitaux Universitaires Mondor, Créteil, France; 6 Bipol Falret, Fondation Falret, St Ouen, France; 7 Clinical Neuroscience, Karolinska Institute, Stockholm, Sweden; 8 Department of Psychiatry, Dalhousie University, Halifax, Canada; 9 Division of Psychiatry, University of Edinburgh, Edinburgh, UK; 10 Department of Child and Adolescent Psychiatry, IISGM, Hospital General Universitario Gregorio Marañón, Madrid, Spain; 11 FIDMAG Research Foundation, Barcelona, Spain; 12 CIBERSAM, Barcelona, Spain; 13 Department of Mental Health and Addiction, University of Oslo, Oslo, Norway; 14 NORMENT K.G. Jebsen Centre for Psychosis Research, Oslo University Hospital, Oslo, Norway; 15 Department of Psychiatry, University of Melbourne, Melbourne, Australia; 16 Department of Psychiatry, University of Münster, Münster, Germany; 17 Division of Neuroscience, San Raffaele Scientific Institute, Milano, Italy; 18 University Vita-Salute San Raffaele, Milano, Italy; 19 Laboratory of Psychiatric Neuroimaging (LIM-21), Departamento e Instituto de Psiquiatria, Hospital das Clinicas HCFMUSP, Faculdade de Medicina, Universidade de São Paulo, São Paulo, Brazil; 20 Centrer for Interdisciplinary Research on Applied Neurosciences (NAPNA), University of São Paulo, São Paulo, Brazil; 21 MRC Centre for Neuropsychiatric Genetics and Genomics, Cardiff University, Cardiff, UK; 22 Interdepartmental Neuroscience Program, University of California, Los Angeles, CA USA; 23 Imaging Genetics Center, Mark and Mary Stevens Neuroimaging and Informatics Institute, Keck School of Medicine of USC, University of Southern California, Marina del Rey, Los Angeles, CA USA; 24 University of Münster, Department of Neurology, Münster, Germany; 25 Department of Psychiatry, University of California San Diego, La Jolla, CA USA; 26 Mental Illness Research Education and Clinical Center, VA San Diego Healthcare System, La Jolla, CA USA; 27 FIDMAG Research Foundation, Barcelona, Spain; 28 CIBERSAM, Barcelona, Spain; 29 Cardiff University Brain Research Imaging Centre (CUBRIC), Cardiff University, Cardiff, UK; 30 Department of Psychiatry and Mental Health, University of Cape Town, Cape Town, South Africa; 31 Neuroscience Institute, University of Cape Town, Cape Town, South Africa; 32 University of Muenster, Institute of Clinical Radiology, Münster, Germany; 33 Department of Psychiatry, Royal Edinburgh Hospital, Edinburgh, UK; 34 Department of Clinical Psychology and Neuropsychology, Johannes Gutenberg-Universität Mainz, Mainz, Germany; 35 Emotion and Development Branch, National Institute of Mental Health, Bethesda, MD USA; 36 Center for Cognitive Ageing and Cognitive Epidemiology, University of Edinburgh, Edinburgh, UK; 37 Psychiatry and Clinical Psychobiology, Division of Neuroscience, Scientific Institute and University Vita-Salute San Raffaele, Milano, Italy; 38 School of Psychiatry, University of New South Wales, Sydney, Australia; 39 Black Dog Institute, Sydney, Sydney, Australia; 40 Univ. Grenoble Alpes, Inserm, U1216, CHU Grenoble Alpes, Grenoble Institut Neurosciences, Grenoble, France; 41 Dept of Psychiatry, SAMRC Unit on Risk & Resilience in Mental Disorders, University of Cape Town, Cape Town, South Africa; 42 Neuroscience Research Australia, Sydney, Australia; 43 School of Medical Sciences, University of New South Wales, Sydney, Australia; 44 West Region, Institute of Mental Health, Singapore, Singapore; 45 Yong Loo Lin School of Medicine, National University of Singapore, Singapore, Singapore; 46 Lee Kong Chian School of Medicine, Nanyang Technological University, Singapore, Singapore; 47 Valkenberg Hospital, Cape Town, South Africa; 48 Institute of Neuroscience, Hospital Clinic, University of Barcelona, IDIBAPS, Barcelona, Spain; 49 Hospital Sírio-Libanês, São Paulo, Brazil; 50 Assistance Publique-Hôpitaux de Paris (AP-HP), CHU Mondor, Psychiatry Department, Créteil, France; 51 Faculté de Médecine, Université Paris Est Créteil, Créteil, France; 52 Institut Pasteur, Unité Perception et Mémoire, Paris, France

**Keywords:** Translational research, Diagnostic markers

## Abstract

Fronto-limbic white matter (WM) abnormalities are assumed to lie at the heart of the pathophysiology of bipolar disorder (BD); however, diffusion tensor imaging (DTI) studies have reported heterogeneous results and it is not clear how the clinical heterogeneity is related to the observed differences. This study aimed to identify WM abnormalities that differentiate patients with BD from healthy controls (HC) in the largest DTI dataset of patients with BD to date, collected via the ENIGMA network. We gathered individual tensor-derived regional metrics from 26 cohorts leading to a sample size of *N* = 3033 (1482 BD and 1551 HC). Mean fractional anisotropy (FA) from 43 regions of interest (ROI) and average whole-brain FA were entered into univariate mega- and meta-analyses to differentiate patients with BD from HC. Mega-analysis revealed significantly lower FA in patients with BD compared with HC in 29 regions, with the highest effect sizes observed within the corpus callosum (*R*^2^ = 0.041, *P*_corr_ < 0.001) and cingulum (right: *R*^2^ = 0.041, left: *R*^2^ = 0.040, *P*_corr_ < 0.001). Lithium medication, later onset and short disease duration were related to higher FA along multiple ROIs. Results of the meta-analysis showed similar effects. We demonstrated widespread WM abnormalities in BD and highlighted that altered WM connectivity within the corpus callosum and the cingulum are strongly associated with BD. These brain abnormalities could represent a biomarker for use in the diagnosis of BD. Interactive three-dimensional visualization of the results is available at www.enigma-viewer.org.

## Introduction

Bipolar disorder (BD) is a severe chronic mental illness that affects ~1% of the general population [1]. There is often a long period with inadequate treatment before the diagnosis is established [2]. Consequently, there is a great need to identify biomarkers of BD. A better understanding of the neurobiology of BD could ultimately help to refine the diagnosis and guide innovative interventions. Recent advances in magnetic resonance imaging (MRI) could help to achieve this goal.

Neural models of BD suggest a role of fronto-limbic dysconnectivity in the emergence of mood symptoms of BD [3, 4]. This model is mainly supported by results from functional MRI (fMRI) studies demonstrating that emotional instability in this disorder might be underpinned by abnormal connectivity between frontal and limbic regions [5, 6]. However, results from diffusion tensor imaging (DTI) studies, a technique that allows the exploration of structural connectivity in vivo, have highlighted far more extensive brain abnormalities in BD. Indeed, the first DTI studies identified alterations in limbic tracts [7–9], followed by numerous studies that reported WM alterations within non-limbic regions, such as the corpus callosum [10–15] and *corona radiata* [16]. Meta-analyses based on whole-brain data have revealed lower fractional anisotropy (FA), a metric derived from DTI known to be positively correlated with the directionality and coherence of white matter bundles [17], in patients with BD near the parahippocampal gyrus, subgenual cingulate cortex [18], temporo-parietal junction and cingulum [19].

Inconsistencies in the location of WM microstructure alterations may be related to limited sample sizes and diversity in methods to collect data from different populations and for DTI data analysis. Indeed, differences in sample characteristics such as age of onset, disease duration, psychotic features, and lithium treatment, all of which have been associated with WM features [12, 20–22], may have contributed to the inconsistency in previous findings. Consequently, large harmonized multi-center studies are required to improve the reliability of case-control findings.

The ENIGMA consortium presents a framework to identify generalizable biomarkers, by analyzing large samples with a harmonized processing pipeline—a strategy that has already identified widespread cortical alterations and specific subcortical volumetric abnormalities in patients with BD [23, 24]. Thus, we analyzed DTI data from the ENIGMA-BD working group with the objectives of (i) identifying reliable generalizable WM abnormalities in BD using mega- and meta- analytics; (ii) testing if clinical characteristics modulate WM microstructure using mega- analytics. Specifically, we expected more pronounced alterations (i.e., larger FA differences with respect to healthy controls) in WM microstructure in patients with a more severe course of illness, and a significant association with psychotropic medication.

## Methods

### Samples

The ENIGMA-BD DTI working group, comprised of 26 cohorts spanning 12 countries, yielded a total of 3033 individuals (1551 healthy controls (HC) and 1482 patients with BD) included in this study. Demographic and clinical information from the whole sample is shown in Table 1; details of the contributing sites may be found in Table S1 and available clinical data for each site is provided in Table S2. Each cohort comprised a minimum of 12 subjects per group and a minimal ratio of patients to controls of 1:3, to allow for robust comparisons and meta-analysis. When needed, we randomly removed some subjects from a given group (mainly control subjects that were too numerous at 4 sites, except for one site that comprised too many patients in comparison to controls; for details, see Table S3). The current analysis includes data acquired until February 2018.

**Table 1:** Descriptive statistics of sample

	Bipolar disorder			Healthy controls				
	*N*^a^	mean/freq^b^	std	*N*^a^	mean/freq^b^	std	*t*/χ^2^	*p*-value
Mean age	1482	39.60	12.15	1551	35.08	12.10	10.11	<0.001
Sex (% females)	1482	60.66%		1551	51.13%		25.77	<0.001
Age of onset	1046	25.19	10.35					
Illness duration	1045	15.47	10.58					
Depression Score								
HDRS-17	115	8.63	8.29					
HDRS-21	285	6.42	8.06					
MADRS	230	9.45	9.79					
Number of depressive episodes	587	5.84	6.02					
Mania Score (YMRS)	545	2.80	3.93					
Number of manic episodes	485	4.30	5.44					
Total number of major episodes	476	10.45	10.15					
Density of episodes^c^	414	0.82	1.00					
On medication	904	84.62%	36.09%					
Antipsychotics	862	45.82%	49.85%					
Antidepressants	903	31.34%	46.41%					
Anticonvulsants	812	39.29%	48.87%					
Lithium	824	42.48%	49.46%					
History of psychotic symptoms	908	54.30%	49.84%					
Lifetime alcohol abuse	272	8.09%	27.32%					
Onset time^d^								
Early	334	31.93%						
Intermediate	534	51.05%						
Late	178	17.02%						
BD type								
BD1	432	77.01%						
BD2	129	22.99%						
Mood phase								
Depressed	313	46.23%						
Euthymic	336	49.63%						
Hypomanic	2	0.30%						
Manic	18	2.66%						
Mixed	8	1.18%						

^a^Number of available data

^b^Proportion calculated among the available data

^c^Density = total episodes/Illness duration

^d^Early: < 18 years ; 18 years < intermediate < 35 years; Late: > 35 years

All participating sites obtained approval from their local ethics committees and all participants gave written informed consent. Participants younger than 18 or older than 65 as well as individuals with diffusion images with low quality after visual inspection (e.g., movement artifacts) were excluded from the analyses.

### Image processing

Acquisition parameters for each of the 26 sites are provided in Table S4. The pre-processing (i.e., eddy current and echo-planar corrections and tensor fitting) was performed at each site using harmonized analysis and quality control protocols from the ENIGMA consortium that have previously been applied in large-scale studies of schizophrenia [25]; recommended pipelines and procedures for the image analyses and quality control are provided online at the ENIGMA-DTI website (http://enigma.ini.usc.edu/protocols/dti-protocols/). After estimation of tensors, each site performed the image analysis and extracted the FA of each region of interest (ROI) (see description in Table S5) according to the ENIGMA-DTI protocol. The multi-subject JHU white matter parcellation atlas [26] was used to parcellate regions of interest from the ENIGMA template in MNI space. Mean FA from 43 regions of interest (ROI) as well as average whole-brain FA were then extracted for each participant across all cohorts.

### Mega-analysis

Our first aim was to identify WM microstructure differences between patients with BD and HC. We merged individual FA values of the 43 ROIs and Average FA (from each cohort) into one mega-analysis and entered them separately in a linear mixed model (using R software version 3.2.1. (R Core Team, 2015) and *lme4* package [27]) including fixed effects for the diagnosis (patients vs. controls), age, sex, and random intercepts for each site:

$$\begin{array}{l}{\mathrm{FA}}\,{\mathrm{ROI}}_i = {\mathrm{Intercept}} + \beta 1 \ast {\mathrm{Diagnosis}} + \beta 2 \ast {\mathrm{Age}} \\ \,\,\,\,\,\,\,\,+ \, \beta 3 \ast {\mathrm{Sex}} + {\mathrm{random}}\,{\mathrm{effect}}\left( {{\mathrm{site}}} \right)\end{array}$$

We used Bonferroni correction to control for multiple comparisons (*p* *<* 0.05/44 = 0.0011). We also assessed the influence of average FA (per subject) across the entire TBSS FA tract skeleton (including core and periphery FA [25]) on local FA differences observed in the first analysis by running the same models including average FA as a covariate.

We performed additional analyses to assess how age, sex, illness duration, age of onset, medication at the time of scan (lithium, antipsychotics, anticonvulsants, and antidepressants), illness severity, history of psychotic symptoms and type of BD (type I vs. type II) might have modulated the main effect of diagnosis. We tested the effect of age and sex by including age-by-diagnosis and sex-by-diagnosis interaction terms. We included medication and history of psychosis as dichotomous measures in the analyses (yes/no variables) and used the density of episodes as an index of illness severity (number of mood episodes/illness duration). Importantly, each analysis controlled for age and sex, so that associations with illness duration and the age of onset would not be confounded by global age differences.

Age, sex, and diagnosis were available for all participants, whereas the remaining variables were available for some sites only (see Table S2 for details of available data for each site).

### Meta-analysis

Given previous demonstrations of the usefulness of meta-analysis for multisite neuroimaging [28], we performed a meta-analysis to allow comparisons with previous ENIGMA studies and comparison across sites. Similarly to previous ENIGMA meta-analyses, we conducted a random-effects inverse-variance weighted meta-analysis (R, metaphore package), to combine Cohen’s *d* effect size of each of the 26 cohorts of the study, both for right and left tracts separately and for bilateral tracts (to allow comparison with other ENIGMA DTI working groups). We calculated the *I*^*2*^ statistic to estimate the heterogeneity of the diagnostic effects across sites. This analysis was run following publicly available scripts on the ENIGMA-GitHub (https://github.com/ENIGMA-git).

## Results

We included 1482 patients with BD and 1551 HC. The patients were significantly older than the controls (mean age BD = 39.6 years; mean age HC = 35.1 years; *t* = 10.11; *p* < 0.001) and comprised a higher proportion of females (60.7 vs. 51.1%; *χ*^2^ = 25.77; *p* < 0.001). We included both age and sex as covariates in the mega- and meta-analyses, and tested for the age-by-diagnosis and sex-by-diagnosis interactions for further exploration of these effects.

### Mega-analysis

**Fig. 1:**
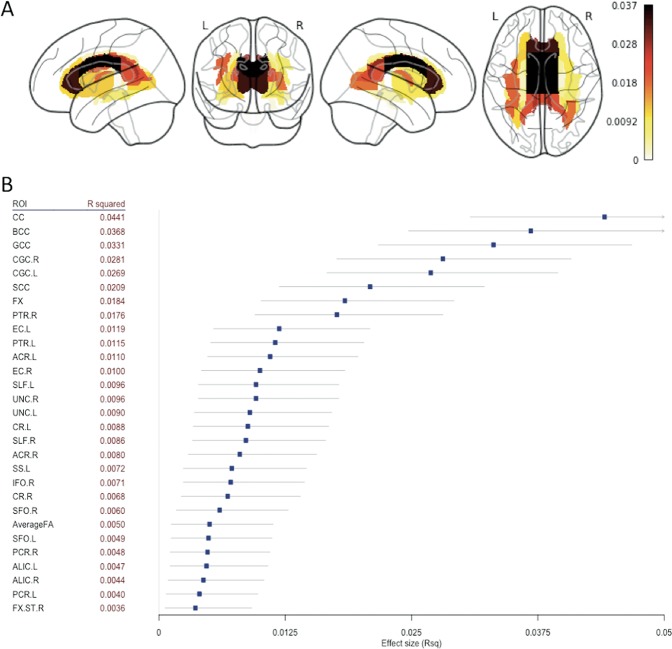
Results of the mega-analysis. **a** Effect sizes of fractional anisotropy (FA) differences between patients with bipolar disorder (BD) and healthy controls projected on the 43 white matter (WM) tracts analyzed. **b** R squared (effect size) with confidence interval, sorted in increasing order of magnitude, for the regions showing significant differences between bipolar patients and healthy controls

Linear mixed models revealed significantly lower FA in BD vs. HC along 29 out of 43 WM tracts and whole skeleton FA (see Table 2, Fig. 1). The largest effect sizes were found in the whole corpus callosum (CC) (*R*^2^ = 0.0441; *P* < 1.0 × 10^−20^), followed by the body (*R*^2^ = 0.0368; *P* < 1.0 × 10^−20^) and genu (*R*^2^ = 0.0331; *P* < 1.0 × 10^−20^) of the CC and the bilateral cinguli (right: *R*^2^ = 0.0281; *P* < 1.0 × 10^−20^; left: *R*^2^ = 0.0269; *P* < 1.0 × 10^−20^). Notably, we found lower FA in bilateral tracts, with the exception of the inferior fronto-occipital fasciculus, where significant difference was observed only in the right hemisphere. In a second analysis, with similar LMM but also covarying for average FA, we still observed lower FA in BD vs. HC across 19 tracts, meaning that the whole-brain average FA moderately influenced the results and that the effects were not exclusively driven by a global decrease in FA in patients (Table S6).

**Table 2:** Mega-analysis results: linear mixed model parameters sorted by effect size (descending order) for FA differences between bipolar patients and healthy controls after controlling for age and sex

ROI	*β*	s.e.	*t*-value	*P*_corr_ > |*t*|	*R*^2^	[0.025	0.975]	Sign.
*Projection fibers*								
PTR.R	0.0098	0.0013	7.3542	1.09E-11	0.0176	0.0095	0.0281	***
PTR.L	0.0079	0.0013	5.9150	1.63E-07	0.0115	0.0051	0.0203	***
ACR.L	0.0065	0.0011	5.8177	2.91E-07	0.0110	0.0048	0.0197	***
CR.L	0.0046	0.0009	5.2310	7.94E-06	0.0088	0.0034	0.0168	***
ACR.R	0.0056	0.0011	4.9348	3.73E-05	0.0080	0.0029	0.0156	***
CR.R	0.0040	0.0009	4.6037	1.90E-04	0.0068	0.0022	0.0140	***
PCR.R	0.0040	0.0011	3.8079	6.29E-03	0.0048	0.0011	0.0110	**
ALIC.L	0.0040	0.0011	3.7958	6.61E-03	0.0047	0.0011	0.0108	**
ALIC.R	0.0038	0.0010	3.6899	1.00E-02	0.0044	0.0009	0.0104	*
PCR.L	0.0036	0.0010	3.4692	2.33E-02	0.0040	0.0007	0.0098	*
SCR.L	0.0028	0.0010	2.7731	2.46E-01	0.0026	0.0002	0.0075	NS
SCR.R	0.0022	0.0010	2.3003	9.46E-01	0.0017	0.0000	0.0060	NS
IC.L	0.0014	0.0008	1.6965	1.00E+00	0.0009	0.0000	0.0044	NS
RLIC.L	0.0014	0.0011	1.3106	1.00E+00	0.0006	0.0000	0.0036	NS
IC.R	0.0011	0.0008	1.3451	1.00E+00	0.0006	0.0000	0.0036	NS
CST.R	−0.0021	0.0016	−1.2829	1.00E+00	0.0005	0.0000	0.0035	NS
CST.L	−0.0017	0.0017	−1.0213	1.00E+00	0.0003	0.0000	0.0030	NS
RLIC.R	0.0009	0.0012	0.7712	1.00E+00	0.0002	0.0000	0.0025	NS
PLIC.L	−0.0007	0.0010	−0.7363	1.00E+00	0.0002	0.0000	0.0024	NS
PLIC.R	−0.0002	0.0010	−0.2474	1.00E+00	0.0000	0.0000	0.0018	NS
*Association fibers*								
CGC.R	0.0136	0.0015	9.3757	0.00E+00	0.0281	0.0176	0.0408	***
CGC.L	0.0138	0.0015	9.1811	0.00E+00	0.0269	0.0166	0.0395	***
EC.L	0.0057	0.0009	6.0965	5.39E-08	0.0119	0.0054	0.0209	***
EC.R	0.0051	0.0009	5.6114	9.65E-07	0.0100	0.0042	0.0184	***
UNC.R	0.0103	0.0019	5.3636	3.87E-06	0.0096	0.0039	0.0178	***
UNC.L	0.0103	0.0020	5.1902	9.87E-06	0.0090	0.0035	0.0171	***
SS.L	0.0054	0.0012	4.6913	1.25E-04	0.0072	0.0024	0.0146	***
IFO.R	0.0080	0.0017	4.6490	1.53E-04	0.0071	0.0024	0.0144	***
SFO.R	0.0057	0.0013	4.2623	9.18E-04	0.0060	0.0017	0.0128	***
SFO.L	0.0053	0.0014	3.8511	5.29E-03	0.0049	0.0012	0.0112	**
FX.ST.R	0.0047	0.0014	3.3251	3.94E-02	0.0036	0.0006	0.0092	*
IFO.L	0.0059	0.0018	3.2375	5.36E-02	0.0035	0.0005	0.0090	NS
SS.R	0.0039	0.0012	3.1308	7.75E-02	0.0032	0.0004	0.0086	NS
FX.ST.L	0.0038	0.0013	2.9044	1.63E-01	0.0028	0.0003	0.0078	NS
CGH.R	0.0038	0.0019	2.0100	1.00E+00	0.0013	0.0000	0.0052	NS
CGH.L	0.0007	0.0018	0.3830	1.00E+00	0.0000	0.0000	0.0019	NS
*Commissural fibers*								
CC	0.0123	0.0010	11.9431	0.00E+00	0.0441	0.0308	0.0594	***
BCC	0.0150	0.0014	10.7856	0.00E+00	0.0368	0.0247	0.0511	***
GCC	0.0123	0.0012	10.2936	0.00E+00	0.0331	0.0217	0.0468	***
SCC	0.0077	0.0009	8.1690	1.95E-14	0.0209	0.0119	0.0322	***
FX	0.0185	0.0025	7.4763	4.42E-12	0.0184	0.0101	0.0292	***
*AverageFA*	0.0025	0.0006	4.0044	2.80E-03	0.0050	0.0012	0.0113	**

*ns* not significant

*p_corr_ < 0.05; **p_corr_ < 0.01; ***p_corr_ < 0.001

#### Age and sex effects

To examine differential effects of age and sex on group differences in FA values, we tested for age-by-diagnosis and sex-by-diagnosis interactions for each ROI. Results showed significant age-by-diagnosis interactions in bilateral superior corona radiata, the posterior limb of the internal capsule and left cingulum, such that there was steeper apparent age-related decline in the HC than BD group in all but the cingulate gyrus portion of the cingulum, where the opposite was found (Table S7; Figure S1). We did not find any significant sex-by-diagnosis interaction (Table S8).

#### Effects of clinical variables

Within the BD group, we found a significant positive relationship of age at onset to FA in the right inferior fronto-occipital fasciculus (Table S9) and a negative association between illness duration and FA within the left cingulum (Table S10) (Fig. S2). In addition, we observed significantly lower FA in patients receiving vs. not receiving antipsychotics within the genu of the CC and in patients receiving vs. not receiving anticonvulsants within multiple ROIs (Figs. S3 and S4; Tables S11 and S12). In contrast, we found higher FA values in several regions among patients receiving vs. not receiving lithium (Fig. S5, Table S13).

We did not observe any significant relationships between FA and antidepressant medication, illness severity, history of psychotic symptoms, or BD subtype (I or II) (see Tables S14–S17).

### Meta-analysis

**Fig. 2:**
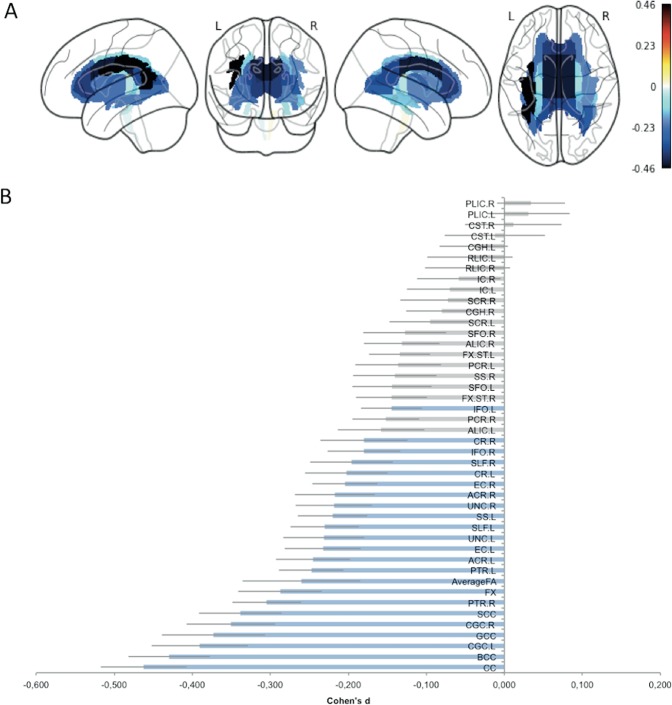
Results of the meta-analysis. **a** Effect sizes for fractional anisotropy (FA) differences between patients with bipolar disorder (BD) and healthy controls projected on the 43 white matter (WM) tracts analyzed. **b** Cohen’s *d* (effect size) sorted in increasing order of magnitude for significant differences between bipolar patients and healthy controls. Significant findings after Bonferroni correction are highlighted in blue. Error bars represent standard error

Results from the meta-analysis revealed lower FA among 23 out of the 44 ROIs (43 tracts and the whole-brain skeleton) analyzed (Table 3, Fig. 2). Similarly to the mega-analysis, the results showed largest effect sizes for the whole CC (*d* = −0.46; *P* = 7.86 × 10^−12^), body of the CC (*d* = −0.43; *P* = 5.41 × 10^−11^), and left cingulum (*d* = −0.39; *P* = 2.38 × 10^−8^). Overall, the meta-analysis showed similar effects to the mega-analysis but was slightly less sensitive. The *I*^*2*^ test indicates small to high heterogeneity across sites for all effect sizes (*I*^*2*^ = 0.002–69.24). To allow comparison with other DTI studies of the ENIGMA consortium, we also conducted a meta-analysis based on bilateral tracts (i.e., 25 ROIs). We found significant decrease FA in patients with BD compared to HC along 15 fasciculi. Similarly, the higher effect sizes were observed for the CC (*d* = −0.46; *P* = 7.86 × 10^−12^) and cingulum (*d* = −0.39; *P* = 4.58 × 10^−8^) (Figure S6, Table S18).

**Table 3:** Meta-analysis results: Cohen’s *d* values, their *s.e*., P-values and I^2^ values (heterogeneity between sites) sorted by effect size (descending order) for FA differences between patients with bipolar disorder and healthy controls after controlling for age and sex

ROI	Cohen’s *d*	s.e.	*p*-value	*I*^2^	*p*-value (corr)	Sign.
*Projection fibers*						
ACR.L	−0.245	0.048	3.14E-07	25.562	8.86E-06	***
ACR.R	−0.217	0.051	2.07E-05	33.382	1.03E-03	**
CR.L	−0.202	0.053	1.32E-04	37.861	4.00E-03	**
CR.R	−0.180	0.056	1.26E-03	43.697	2.60E-02	*
ALIC.L	−0.158	0.056	4.41E-03	43.311	2.32E-01	NS
PCR.R	−0.152	0.043	4.26E-04	12.031	1.13E-01	NS
PCR.L	−0.136	0.055	1.37E-02	42.736	7.39E-01	NS
ALIC.R	−0.131	0.048	6.52E-03	26.872	2.78E-01	NS
SCR.L	−0.095	0.052	6.90E-02	36.840	1.00E+00	NS
SCR.R	−0.072	0.061	2.36E-01	53.242	1.00E+00	NS
IC.L	−0.070	0.055	2.01E-01	41.932	1.00E+00	NS
IC.R	−0.058	0.054	2.75E-01	39.437	1.00E+00	NS
RLIC.R	−0.047	0.055	3.90E-01	41.619	1.00E+00	NS
RLIC.L	−0.044	0.055	4.21E-01	41.598	1.00E+00	NS
CST.L	−0.012	0.065	8.49E-01	58.035	1.00E+00	NS
CST.R	0.012	0.062	8.50E-01	53.890	1.00E+00	NS
PLIC.L	0.031	0.053	5.60E-01	37.920	1.00E+00	NS
PLIC.R	0.034	0.044	4.35E-01	13.722	1.00E+00	NS
*Association fibers*						
CGC.L	−0.391	0.062	2.99E-10	53.489	2.38E-08	***
CGC.R	−0.350	0.057	7.50E-10	45.173	2.00E-06	***
EC.L	−0.233	0.049	1.65E-06	27.203	1.06E-04	***
UNC.L	−0.231	0.052	8.16E-06	35.172	3.30E-04	***
SS.L	−0.220	0.044	6.96E-07	15.563	5.59E-05	***
UNC.R	−0.219	0.049	8.05E-06	28.252	1.30E-03	**
EC.R	−0.205	0.042	1.09E-06	8.287	2.58E-04	***
IFO.R	−0.180	0.047	1.19E-04	22.441	3.99E-03	**
IFO.L	−0.145	0.039	2.24E-04	0.000	1.41E-02	*
FX.ST.R	−0.145	0.045	1.43E-03	18.610	9.68E-02	NS
SFO.L	−0.144	0.051	4.67E-03	33.405	2.00E-01	NS
SS.R	−0.140	0.053	8.63E-03	39.037	3.17E-01	NS
FX.ST.L	−0.134	0.039	6.40E-04	0.002	5.12E-02	NS
SFO.R	−0.127	0.053	1.65E-02	38.320	7.26E-01	NS
CGH.R	−0.080	0.045	7.69E-02	18.794	1.00E+00	NS
CGH.L	−0.039	0.044	3.71E-01	14.618	1.00E+00	NS
*Commissural fibers*						
CC	−0.462	0.055	5.08E-17	41.305	7.86E-12	***
BCC	−0.430	0.052	2.32E-16	35.479	5.41E-11	***
GCC	−0.373	0.066	1.78E-08	59.395	6.87E-06	***
SCC	−0.339	0.053	1.97E-10	37.906	5.66E-08	***
FX	−0.288	0.054	8.19E-08	39.029	7.84E-05	***
*AverageFA*	−0.260	0.076	5.69E-04	69.240	1.66E-01	NS

*ns* not significant

*p_corr_ < 0.05; **p_corr_ < 0.01; ***p_corr_ < 0.001

## Discussion

In the largest multi-center DTI study of BD to date, we found alterations of WM microstructure in patients with BD along multiple bundles, with strongest effects within the CC and the cingulum. FA was lower in patients in most ROIs, although effect sizes were small. Age, age of onset, illness duration as well as anticonvulsants and antipsychotic medications were associated with lower FA.

We collected individual data from 1482 patients and 1551 controls across 26 international cohorts, allowing a sample size considerably exceeding all prior DTI studies of BD. Unlike most studies that found localized WM alterations in BD, we identified widespread abnormalities (lower FA along 29 out of the 44 regions analyzed in the mega-analysis and 32 out of 44 ROIs in the meta-analysis). Similarly to results in the ENIGMA DTI schizophrenia project, this suggests a global profile of microstructural abnormalities in BD, which are however not specific to that disorder [25].

For both analyses (i.e., mega and meta), the largest effect sizes were observed within the CC and cingulum. This is consistent with a recent meta-analysis showing decreased FA within the CC, cingulum and the anterior superior longitudinal fasciculus in BD in comparison to controls [29]. The cingulum is a major pathway in the limbic system. Impairment of cingulum and uncinate structural integrity is in good agreement with previous models of altered fronto-limbic connectivity in BD [3, 30].

In contrast, the role of the CC in pathophysiological models of BD is less straightforward. Disconnection in patients with BD with psychotic history has been suggested [12] but there is no clear evidence for the implication of the CC in emotion processing or mood switching [31]. Reduced FA within the CC was also reported in a meta-analysis of DTI studies in schizophrenia [25] and major depressive disorder [29], suggesting an overlapping involvement in both psychosis and affective disorders. Further studies are warranted to evaluate to what extent the CC is differentially affected in these disorders. Preliminary data suggest that disruption of interhemispheric connectivity is a disease marker rather than a vulnerability marker to BD [32]. Nonetheless, we identified extensive WM abnormalities suggesting that current pathophysiological models of BD are incomplete. Future models should not be limited to fronto-limbic networks, and should perhaps consider interhemispheric disconnectivity as a key feature of BD.

Importantly, the patient group was significantly older than the control group. Although we controlled for age in all analyses, it is possible that the linear models used are not fully accounting for the age-related variance [33]. However, the assessment of the effects of age revealed a significant interaction between age and diagnosis for only 4 ROIs out of the 43 analyzed. We found a significant increase in the effect of age in patients with BD for the left CGC only, while we found the reverse association for the bilateral SCR and the left PLIC, these effects were not anticipated and should be verified when replication samples become available.

We found that lithium intake was associated with higher FA in several tracts, as well as with global FA. Prior studies have suggested neuroprotective effects of lithium, on gray matter [23, 34–36] and white matter [37]. Higher FA associated with lithium use could reflect a direct influence of lithium on water diffusion or a beneficial effect on myelination [38], as suggested by the observation that lithium promotes myelin gene expression, morphological maturation, and remyelination in cultured oligodendrocytes via the Wnt/β-catenin and the Akt/CREB pathways [39]. In patients with BD, lithium may increase axial diffusivity in WM tracts also influenced by genetic variation in this pathway [22]. We also found lower FA in patients who received anticonvulsants in several tracts and average global FA. Further, patients who were on antipsychotic treatment showed lower FA within the genu of the CC. This is consistent with prior results suggesting a negative relationship between anticonvulsants, antipsychotics and cortical thickness or FA [23, 37]. However, it could be possible that the choice of the medication was driven by some patients’ particularities or unknown neurobiological characteristics, which are hard to assess with a cross-sectional design, leading to confounding by indication. Longitudinal clinical trials are needed to clarify this point.

We did not find significant differences between BD type I and type II. The power of prior meta-analyses of DTI studies has also been too low to perform this comparison. However, sensitivity analyses for these meta-analyses indicated that the sub-group of patients with BD I was driving the FA difference observed between patients with BD and HC [19, 29, 40]. Although we had enough power, the comparison of BD I vs. BD II did not replicate this result. Consistent with our results, however, ENIGMA analyses of T1-weighted anatomical MRI data of patients with BD did not yield any detectable differences between BD types [23, 24].

In sum, the multisite nature of the study is a strength that allowed us to detect small but significant differences. Our results seem to challenge the hypothesis of a precise localization for the WM alterations in BD. Indeed, we have highlighted extensive abnormalities, which do not seem to be specific to this psychiatric disorder. Lower FA across multiple bundles has already been consistently observed in studies of schizophrenia, with apparently higher effect sizes (e.g., [25]). Consequently, to build more precise neurobiological models of BD future studies should benefit from new advanced neuroimaging methods such as Neurite Orientation Dispersion and Density Imaging (NODDI) [41]. This recent processing model allows fine-grained measurement of the WM microstructure, with physiological interpretation of the derived indices, and has already shown promising results in BD [42]. However, the large-scale application of such methods will only be possible with raw data sharing within international consortia. This will allow the application of advanced DTI models and whole-brain analyses, which are needed to better understand WM abnormalities observed in BD. Finally, longitudinal studies conducted in conjunction with advanced DTI protocols are essential to clarify the impact of pharmaceutical treatments on brain microstructure.

Some limitations are important to emphasize. We did not include other diffusion parameters in our analysis. Lower FA may represent abnormal fiber coherence but does not yield information on fiber density or myelination. The mean, radial and axial diffusivity measure would have added complementary information regarding the nature of WM alteration. However, we have focused on the most commonly used measure, which offers better comparability with prior studies. Also, most studies have highlighted a correlation between FA and these other measures, while their inclusion would have tripled the number of analyses. In addition, although we found “widespread” WM abnormalities in patients with BD, the robust ENIGMA DTI pipeline used to partition the ROIs involved only long and isolinear bundles. With this methodological approach (i.e., FSL TBSS), we cannot evaluate localized changes within the superficial WM, as have been previously observed in BD and schizophrenia [43]. Also, this methodological approach poorly reconstructs fiber crossings, which may have led to incomplete localization of group differences. Further studies are warranted to identify more fine-grained WM abnormalities in BD.

Importantly, retrospective multisite analyses have some limitations. Differences in the acquisition parameters, magnet strength, head coil and manufacturer provided software could have impacted the results. However, we believe that our approach, using a harmonized data processing pipeline, with a reliable procedure, allows for the first time coordinated mega- and meta-analyses with robust results.

Moreover, the effects of the covariates found here are only derived from post hoc analyses in cross-sectional studies with a somewhat limited representation of individuals with BD over age 50 (only 18% of the sample). Longitudinal studies would be more suitable to identify and predict the effect of age, illness duration/severity and medication on WM microstructure in patients with BD. In addition, despite their importance, we were not able to test the relation between FA and other covariates, such as body mass index and frequent BD comorbidities (e.g., anxiety or substance use disorder). Too few sites had collected these measures to allow robust analyses. However, we believe that our sample is ecologically valid and captures the heterogeneity of BD.

With this unprecedented sample size, we found evidence for widespread WM abnormalities in patients with BD and showed differences in BD WM microstructure that were unobserved until now. These results may inform future DTI studies with regard to expected effect sizes, and the effects of several covariates and clinical variables. We also highlighted that the CC and the cingulum had the strongest decrease in FA in patients with BD. Despite growing evidence for altered structure of the CC in BD, its specific role in the pathophysiology of BD needs to be further integrated into neural models of BD.

## Funding and disclosure

The researchers and studies included in this paper were funded by the: German Research Foundation (DFG, grant FOR2107 DA1151/5-1 and DA1151/5-2 to UD; SFB-TRR58, Projects C09 and Z02 to UD) and the Interdisciplinary Center for Clinical Research (IZKF) of the medical faculty of Münster (grant Dan3/012/17 to UD); German Research Foundation (SFB636/C6, WE3638/3-1); Oslo University Hospital, University of Oslo, Norwegian Research Council, South Eastern Norwegian Health Authorities; NIH Grant MH083968; VA Desert-Pacific Mental Illness Research Education and Clinical Center; Research Council of Norway (223273, 213837, 249711, 249795, 248238, 248778, and 262656), South East Norway Health Authority (2017-112) Kristian Gerhard Jebsen Stiftelsen (SKGJ-MED-008) and the European Community's Seventh Framework Program (FP7/2007–2013), grant agreement no. 602450 (IMAGEMEND); Conselho Nacional de Desenvolvimento Científico e Tecnológico (CNPq, Brazil, 480370/2009-5); Australian National Medical and Health Research Council (Program Grant 1037196), and the Lansdowne Foundation; NIH grant U54 EB020403 from the BD2K Initiative, R01 MH116147, and P41 EB015922; CIBERSAM; NHG (SIG/12004) and SBIC (RP C-009) (KS); SAMRC (DJS); Canadian Institutes of Health Research (103703, 106469 and 64410); Nova Scotia Health Research Foundation, Dalhousie Clinical Research and Scholarship to T Hajek, NARSAD Young Investigator and Independent Investigator Awards (TH), JMAS SIM fellowship from the Royal College of Physicians of Edinburgh and an ESAT College Fellowship from the University of Edinburgh (HCW); Part of the Cardiff cohort was funded through a NARSAD Young Investigator Award (17319) (XC), we are also grateful to the National Centre for Mental Health (NCMH) and the Bipolar Disorder Research Network for their support with recruitment; Spanish Ministry of Science, Innovation and Universities (PI15/00283) integrated into the Plan Nacional de I+D+I y cofinanciado por el ISCIII-Subdirección General de Evaluación y el Fondo Europeo de Desarrollo Regional (FEDER), CIBERSAM, and the Comissionat per a Universitats i Recerca del DIUE de la Generalitat de Catalunya to the Bipolar Disorders Group (2017 SGR 1365) and the project SLT006/17/00357, from PERIS 2016-2020 (Departament de Salut). CERCA Programme/Generalitat de Catalunya (EV); FAPESP-Brazil (#2009/14891-9, 2010/18672-7, 2012/23796-2 and 2013/03905-4), CNPq-Brazil (#478466/2009 and 480370/2009), the Wellcome Trust (UK) and the Brain & Behavior Research Foundation (NARSAD Independent Investigator Award (GFB); CRKC was supported by NIA T32AG058507; NIH/NIMH 5T32MH073526 and NIH grant U54EB020403 from the Big Data to Knowledge (BD2K) Program. CRKC has received partial research support from Biogen, Inc. (Boston, USA) for work unrelated to the topic of this manuscript; the Human Brain Project, funded from the European Union’s Horizon 2020 Framework Program for Research and Innovation under the Specific Grant Agreements No. 785907 (SGA2) and No: 604102 (SGA1), and by the FRM DIC20161236445; IRMaGe MRI/Neurophysiology facility which was partly funded by the French program “Investissement d’Avenir” run by the “Agence Nationale pour la Recherche”; grant “Infrastructure d’avenir en Biologie Santé”—ANR-11-INBS-0006” and the Agence Nationale pour la Recherche (ANR-11-IDEX-0004 Labex BioPsy, ANR-10-COHO-10-01 psyCOH), Fondation pour la Recherche Médicale (Bioinformatique pour la biologie 2014) and the Fondation de l'Avenir (Recherche Médicale Appliquée 2014). PMT and NJ received research grant from Biogen, Inc., for research unrelated to this manuscript. Dr. Vieta has received grants and served as consultant, advisor or CME speaker for the following entities: AB-Biotics, Abbott, Allergan, Angelini, Dainippon Sumitomo Pharma, Galenica, Janssen, Lundbeck, Novartis, Otsuka, Sage, Sanofi-Aventis, and Takeda. OAA has received speakers honorarium from Lundbeck, and is consultant for HealthLytix. DJS has received research grants and/or consultancy honoraria from Lundbeck and Sun. The remaining authors declare no competing interests.

## Supplementary information

Supplemental Material
