# Correction: Widespread white matter microstructural abnormalities in bipolar disorder: evidence from mega- and meta-analyses across 3033 individuals

**DOI:** 10.1038/s41386-019-0521-6

**Published:** 2019-09-16

**Authors:** Pauline Favre, Melissa Pauling, Jacques Stout, Franz Hozer, Samuel Sarrazin, Christoph Abé, Martin Alda, Clara Alloza, Silvia Alonso-Lana, Ole A. Andreassen, Bernhard T. Baune, Francesco Benedetti, Geraldo F. Busatto, Erick J. Canales-Rodríguez, Xavier Caseras, Tiffany Moukbel Chaim-Avancini, Christopher R. K. Ching, Udo Dannlowski, Michael Deppe, Lisa T. Eyler, Mar Fatjo-Vilas, Sonya F. Foley, Dominik Grotegerd, Tomas Hajek, Unn K. Haukvik, Fleur M. Howells, Neda Jahanshad, Harald Kugel, Trine V. Lagerberg, Stephen M. Lawrie, Julia O. Linke, Andrew McIntosh, Elisa M. T. Melloni, Philip B. Mitchell, Mircea Polosan, Edith Pomarol-Clotet, Jonathan Repple, Gloria Roberts, Annerine Roos, Pedro G. P. Rosa, Raymond Salvador, Salvador Sarró, Peter R. Schofield, Mauricio H. Serpa, Kang Sim, Dan J. Stein, Jess E. Sussmann, Henk S. Temmingh, Paul M. Thompson, Norma Verdolini, Eduard Vieta, Michele Wessa, Heather C. Whalley, Marcus V. Zanetti, Marion Leboyer, Jean-François Mangin, Chantal Henry, Edouard Duchesnay, Josselin Houenou, for the ENIGMA Bipolar Disorder Working Group

**Affiliations:** 1 Neurospin, CEA, Université Paris-Saclay, Gif-sur-Yvette, France; 2 INSERM Unit U955, Team 15, “Translational Psychiatry”, Créteil, France; 3 Assistance Publique-Hôpitaux de Paris (AP-HP), Corentin-Celton Hospital, Department of Psychiatry, Issy-les-Moulineaux, France; 4 Paris Descartes University, PRES Sorbonne Paris Cité, Paris, France; 5 Pôle de psychiatrie, DHU PePSY, Hôpitaux Universitaires Mondor, Créteil, France; 6 Bipol Falret, Fondation Falret, St Ouen, France; 7 Clinical Neuroscience, Karolinska Institute, Stockholm, Sweden; 8 Department of Psychiatry, Dalhousie University, Halifax, Canada; 9 Division of Psychiatry, University of Edinburgh, Edinburgh, UK; 10 Department of Child and Adolescent Psychiatry, IISGM, Hospital General Universitario Gregorio Marañón, Madrid, Spain; 11 FIDMAG Research Foundation, Barcelona, Spain; 12 CIBERSAM, Barcelona, Spain; 13 Department of Mental Health and Addiction, University of Oslo, Oslo, Norway; 14 NORMENT K.G. Jebsen Centre for Psychosis Research, Oslo University Hospital, Oslo, Norway; 15 Department of Psychiatry, University of Melbourne, Melbourne, Australia; 16 Department of Psychiatry, University of Münster, Münster, Germany; 17 Division of Neuroscience, San Raffaele Scientific Institute, Milano, Italy; 18 University Vita-Salute San Raffaele, Milano, Italy; 19 Laboratory of Psychiatric Neuroimaging (LIM-21), Departamento e Instituto de Psiquiatria, Hospital das Clinicas HCFMUSP, Faculdade de Medicina, Universidade de São Paulo, São Paulo, Brazil; 20 Centrer for Interdisciplinary Research on Applied Neurosciences (NAPNA), University of São Paulo, São Paulo, Brazil; 21 MRC Centre for Neuropsychiatric Genetics and Genomics, Cardiff University, Cardiff, UK; 22 Interdepartmental Neuroscience Program, University of California, Los Angeles, CA USA; 23 Imaging Genetics Center, Mark and Mary Stevens Neuroimaging and Informatics Institute, Keck School of Medicine of USC, University of Southern California, Marina del Rey, Los Angeles, CA USA; 24 University of Münster, Department of Neurology, Münster, Germany; 25 Department of Psychiatry, University of California San Diego, La Jolla, CA USA; 26 Mental Illness Research Education and Clinical Center, VA San Diego Healthcare System, La Jolla, CA USA; 27 FIDMAG Research Foundation, Barcelona, Spain; 28 CIBERSAM, Barcelona, Spain; 29 Cardiff University Brain Research Imaging Centre (CUBRIC), Cardiff University, Cardiff, UK; 30 Department of Psychiatry and Mental Health, University of Cape Town, Cape Town, South Africa; 31 Neuroscience Institute, University of Cape Town, Cape Town, South Africa; 32 University of Muenster, Institute of Clinical Radiology, Münster, Germany; 33 Department of Psychiatry, Royal Edinburgh Hospital, Edinburgh, UK; 34 Department of Clinical Psychology and Neuropsychology, Johannes Gutenberg-Universität Mainz, Mainz, Germany; 35 Emotion and Development Branch, National Institute of Mental Health, Bethesda, MD USA; 36 Center for Cognitive Ageing and Cognitive Epidemiology, University of Edinburgh, Edinburgh, UK; 37 Psychiatry and Clinical Psychobiology, Division of Neuroscience, Scientific Institute and University Vita-Salute San Raffaele, Milano, Italy; 38 School of Psychiatry, University of New South Wales, Sydney, Australia; 39 Black Dog Institute, Sydney, Sydney, Australia; 40 Univ. Grenoble Alpes, Inserm, U1216, CHU Grenoble Alpes, Grenoble Institut Neurosciences, Grenoble, France; 41 Dept of Psychiatry, SAMRC Unit on Risk & Resilience in Mental Disorders, University of Cape Town, Cape Town, South Africa; 42 Neuroscience Research Australia, Sydney, Australia; 43 School of Medical Sciences, University of New South Wales, Sydney, Australia; 44 West Region, Institute of Mental Health, Singapore, Singapore; 45 Yong Loo Lin School of Medicine, National University of Singapore, Singapore, Singapore; 46 Lee Kong Chian School of Medicine, Nanyang Technological University, Singapore, Singapore; 47 Valkenberg Hospital, Cape Town, South Africa; 48 Institute of Neuroscience, Hospital Clinic, University of Barcelona, IDIBAPS, Barcelona, Spain; 49 Hospital Sírio-Libanês, São Paulo, Brazil; 50 Assistance Publique-Hôpitaux de Paris (AP-HP), CHU Mondor, Psychiatry Department, Créteil, France; 51 Faculté de Médecine, Université Paris Est Créteil, Créteil, France; 52 Institut Pasteur, Unité Perception et Mémoire, Paris, France

**Keywords:** Translational research, Diagnostic markers

**Correction to:** *Neuropsychopharmacology* 10.1038/s41386-019-0485-6, published online 21 August 2019.

This Article was originally published under NPG's License to Publish, but has now been made available under a [CC BY 4.0] license. The PDF and HTML versions of the Article have been modified accordingly.

