# Supplementary material for: Widespread white matter microstructural abnormalities in bipolar disorder: evidence from mega- and meta-analyses across 3033 individuals
Source: Neuropsychopharmacology. 2019 Aug 21;44(13):2285–93. doi: 10.1038/s41386-019-0485-6 (PMC6898371; doi:10.1038/s41386-019-0485-6)
Supplement: Supplementary file 1 — Supplemental Material [file 41386_2019_485_MOESM1_ESM.docx]

Supplemental Material

**Content**

## Figure S1. Relationship of FA values to age and Diagnosis

Figure S2. Relationship of FA values to age of illness onset and illness duration

Figure S3. Decreased FA in patients with BD associated with antipsychotic treatment

Figure S4. Decreased FA in patients with BD associated with anticonvulsant treatment

Figure S5. Increased FA in patients with BD associated with lithium treatment

Figure S6. Results of the meta-analysis based on bilateral tracts

## Table S1. Demographic details of the contributing sites

## Table S2. Available clinical data for each site

Table S3. Demographic details of the original samples (resampled sites)

## Table S4. Diffusion tensor imaging acquisitions parameters for each site

Table S5. Description of the ROIs

Table S6. Mega-analysis: Linear mixed model results for FA differences between bipolar patients and healthy controls after controlling for average FA. age and sex

Table S7. Mega-analysis: Linear mixed model results for the interaction between diagnosis and age after controlling for sex

Table S8. Mega-analysis: Linear mixed model results for the interaction between diagnosis and sex after controlling for age

Table S9. Mega-analysis: Linear mixed model results for the main effect of the age of onset after controlling for age and sex

Table S10. Mega-analysis: Linear mixed model results for the main effect of illness duration after controlling for age and sex

Table S11. Mega-analysis: Linear mixed model results for the main effect of antipsychotic treatment after controlling for age and sex

Table S12. Mega-analysis: Linear mixed model results for the main effect of anticonvulsant treatment after controlling for age and sex

Table S13. Mega-analysis: Linear mixed model results for the main effect of lithium treatment after controlling for age and sex

Table S14. Mega-analysis: Linear mixed model results for the main effect of antidepressant treatment after controlling for age and sex

Table S15. Mega-analysis: Linear mixed model results for the main effect of the severity of the disease (density of episodes) after controlling for age and sex

Table S16. Mega-analysis: Linear mixed model results for the main effect of history of psychotic symptoms after controlling for age and sex

Table S17. Mega-analysis: Linear mixed model results for the main effect of type of bipolar disorder after controlling for age and sex

Table S18. Meta-analysis on bilateral tracts: Cohen’s *d* values, their *s.e*., P-values and I^2^ values (heterogeneity between sites) sorted by effect size (descending order) for FA differences between patients with bipolar disorder and healthy controls after controlling for age and sex.

# Supplemental Figures


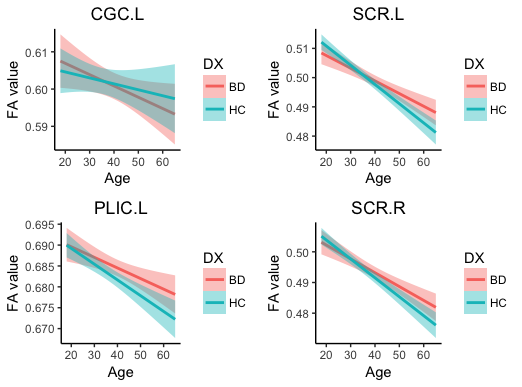


### Figure S1. Relationship of FA values to age and diagnosis (BD = Bipolar disorder; CGC.L = left cingulum; HC = Healthy controls; PLIC.L = left posterior limb of the internal capsule; SCR.R/L = superior corona radiata left/right)


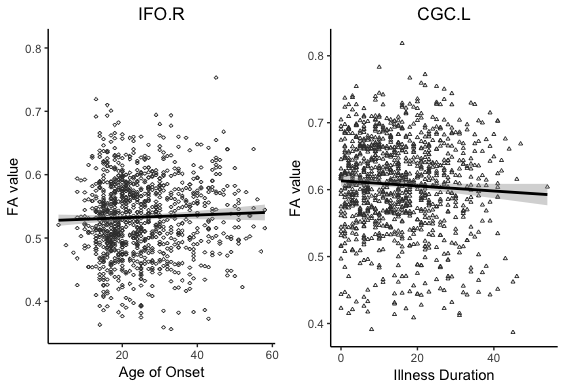


### Figure S2. Relationship of FA values to age of illness onset and illness duration (IFO.R = right inferior fronto-occipital fasciculus; CGC.L = left cingulum)

###
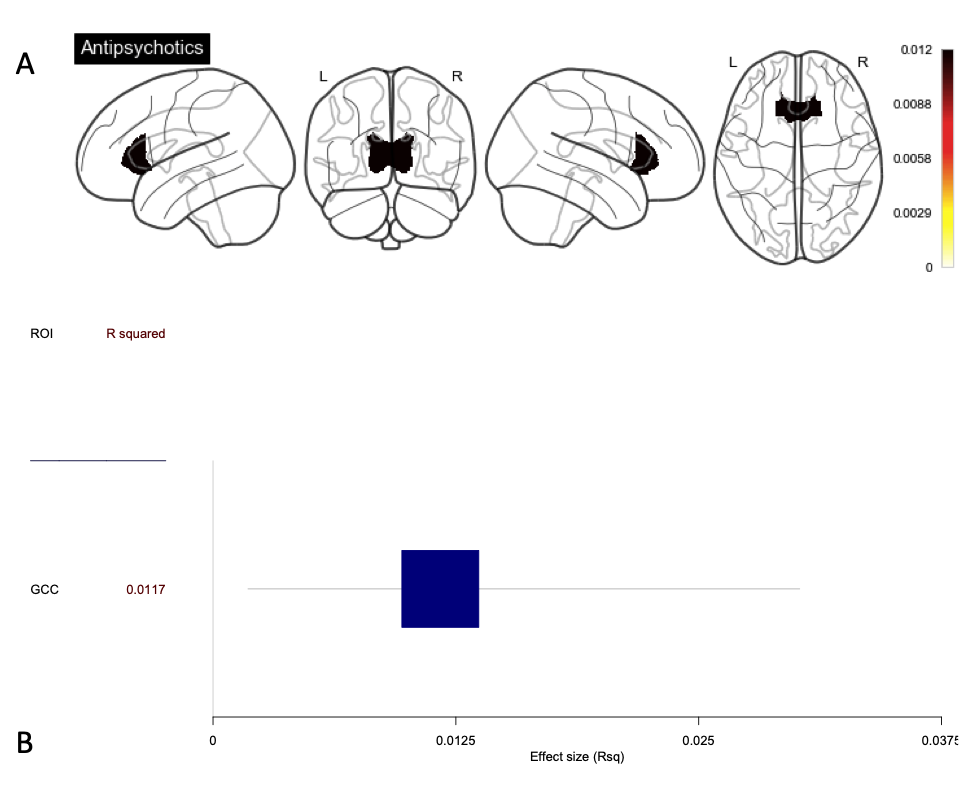


### Figure S3. Decreased FA in patients with BD associated with antipsychotic treatment. A) Effect sizes of fractional anisotropy (FA) differences between patients ON vs. OFF Antipsychotic treatment projected on white matter (WM) tracts showing significant difference. B) R squared effect size with confidence interval sorted in increase magnitude for the regions showing significant differences between patients ON vs. OFF Antipsychotics.

###
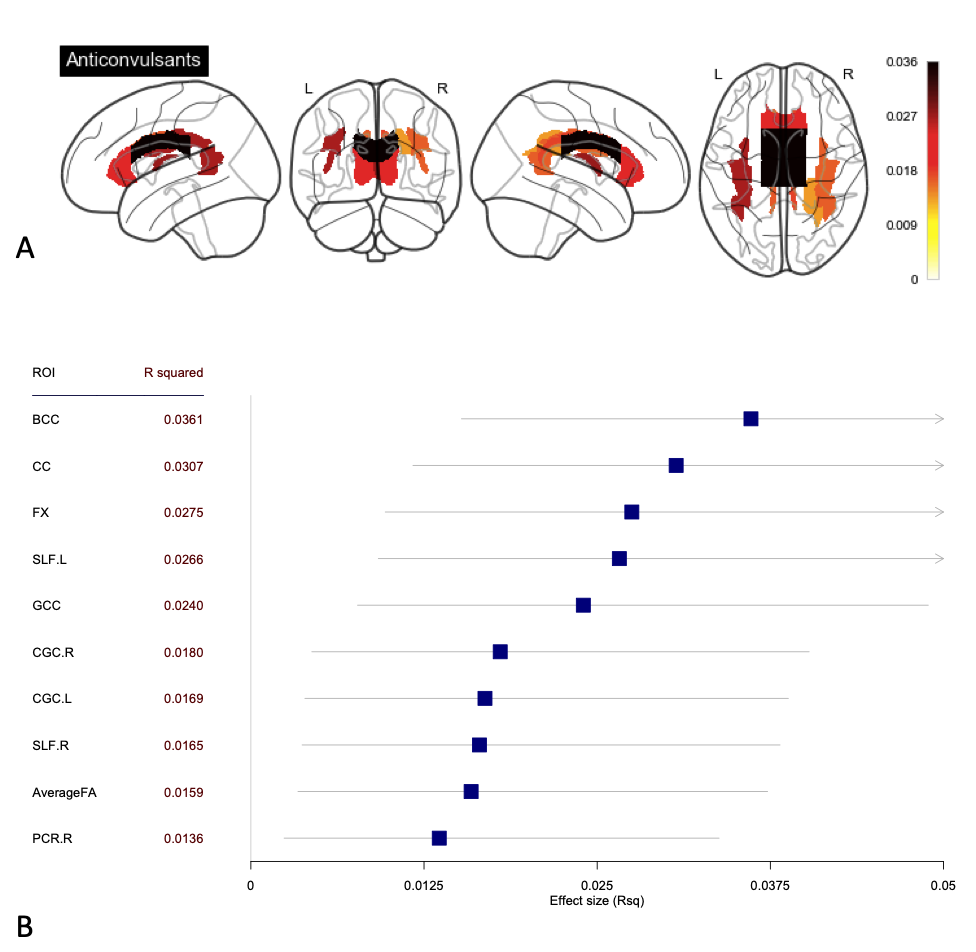


### Figure S4. Decreased FA in patients with BD associated with anticonvulsant treatment. A) Effect sizes of fractional anisotropy (FA) differences between patients ON vs. OFF Anticonvulsant treatment projected on white matter (WM) tracts showing significant difference. B) R squared effect size with confidence interval sorted in increase magnitude for the regions showing significant differences between patients ON vs. OFF Anticonvulsants

###
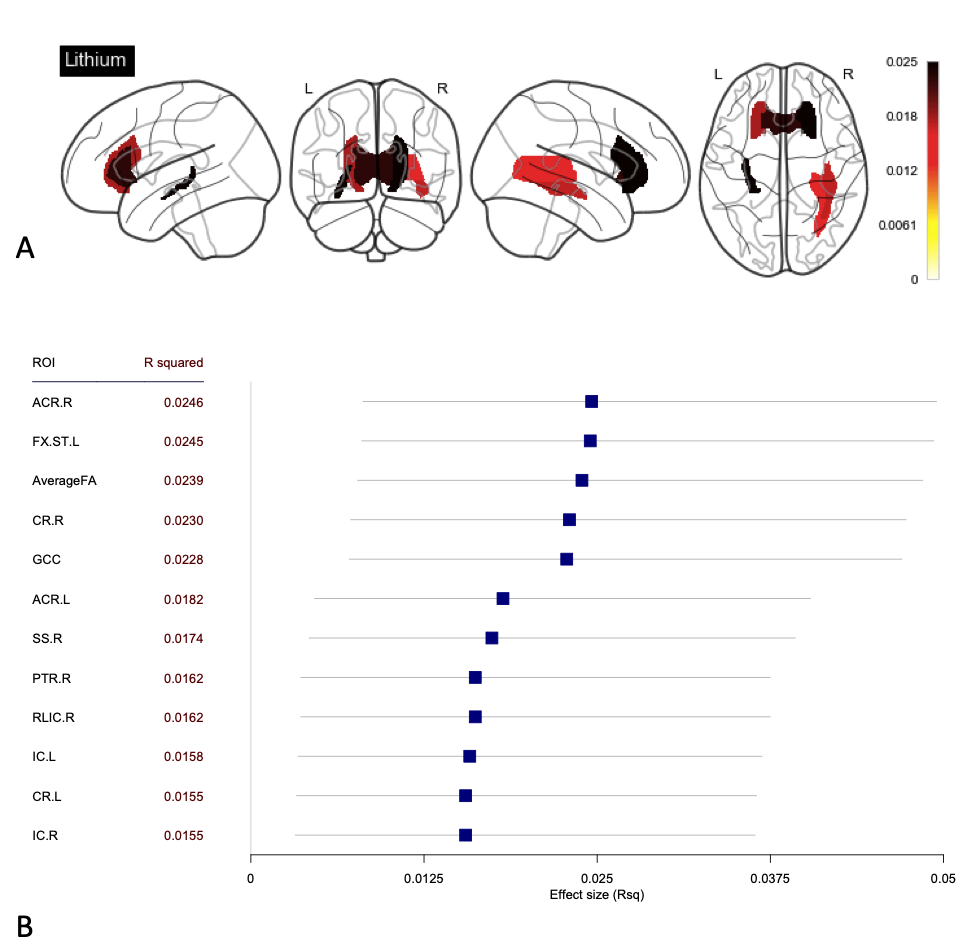


### Figure S5. Increased FA in patients with BD associated with lithium treatment. A) Effect sizes of fractional anisotropy (FA) differences between patients ON vs. OFF Lithium treatment projected on white matter (WM) tracts showing significant difference. B) R squared effect size with confidence interval sorted in increase magnitude for the regions showing significant differences between patients ON vs. OFF Lithium.

###
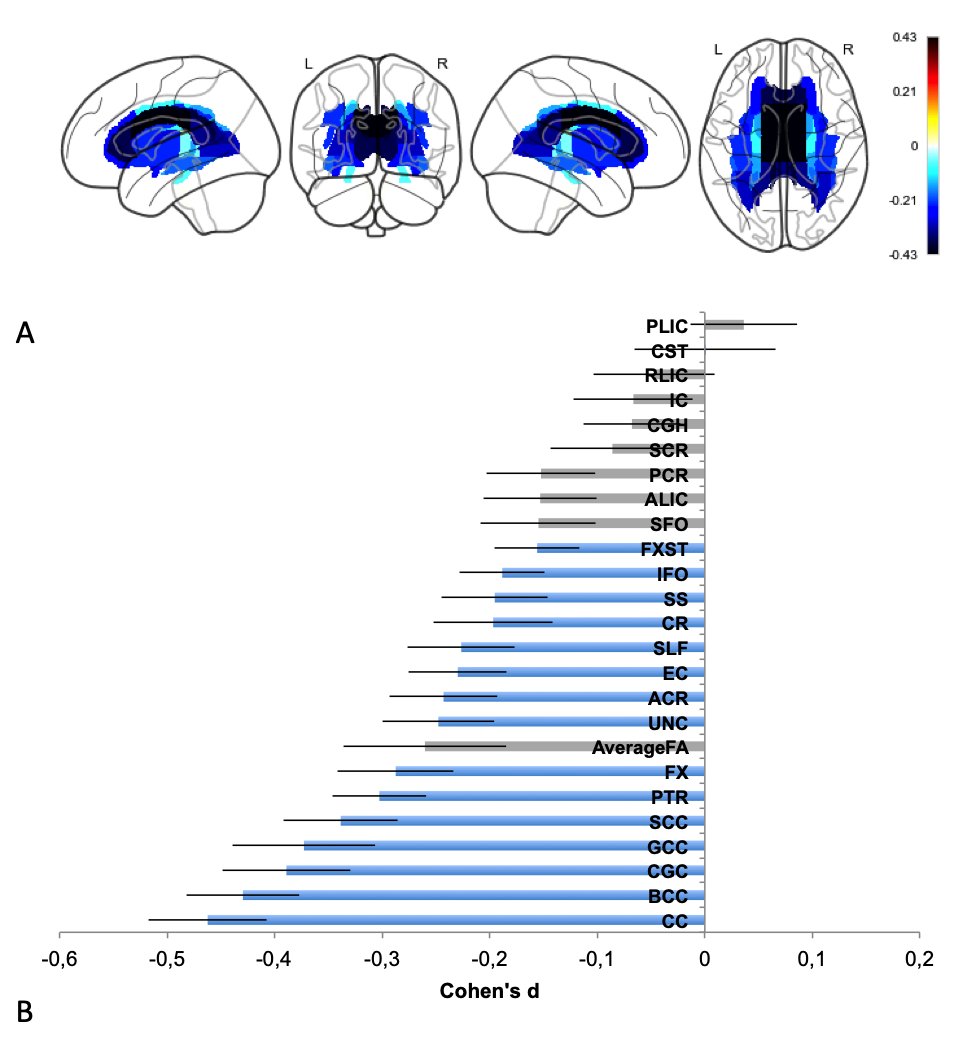


### Figure S6. Results of the meta-analysis based on bilateral tracts. A) Effect sizes of fractional anisotropy (FA) differences between patients with bipolar disorder (BD) and healthy controls projected on the 25 white matter (WM) tracts analyzed. B) Cohen’s d effect size sorted in increase magnitude for significant differences between bipolar patients and healthy controls. Significant findings after Bonferroni correction are highlighted in blue. Error bars represent standard error.

# Supplemental Tables

## Table S1. Demographic details of the contributing sites

| **Cohort** | **Bipolar Patients** | | | **Healthy Controls** | | |  |
| --- | --- | --- | --- | --- | --- | --- | --- |
|  | N | Age | %F | N | Age | %F | N Tot. |
| 1_Munster | 41 | 39.0 | 51.2% | 122 | 37.9 | 53.3% | 163 |
| 2_UNSW | 61 | 25.3 | 68.9% | 80 | 22.7 | 53.8% | 141 |
| 3.1_UNC | 60 | 33.9 | 60.0% | 42 | 33.3 | 50.0% | 102 |
| 3.2_UNC | 20 | 34.5 | 35.0% | 9 | 35.2 | 11.1% | 29 |
| 4_UCT | 20 | 29.8 | 55.0% | 24 | 26.0 | 45.8% | 44 |
| 5_KI | 38 | 41.3 | 65.8% | 12 | 42.5 | 25.0% | 50 |
| 6_Cardiff | 63 | 43.8 | 63.5% | 40 | 43.5 | 60.0% | 103 |
| 7.1_Edimburgh | 12 | 44.8 | 33.3% | 34 | 36.9 | 47.1% | 46 |
| 7.2_Edimburgh | 27 | 46.3 | 63.0% | 24 | 43.8 | 58.3% | 51 |
| 8.1_IoL | 35 | 35.1 | 51.4% | 104 | 33.1 | 40.4% | 139 |
| 8.2_IoL | 18 | 36.1 | 72.2% | 57 | 25.0 | 49.1% | 75 |
| 8.3_IoL | 84 | 32.2 | 72.6% | 96 | 32.2 | 62.5% | 180 |
| 9.1_Oslo_TOP | 57 | 31.5 | 50.9% | 174 | 32.4 | 41.4% | 231 |
| 9.2_Oslo_Malt | 42 | 36.0 | 73.8% | 41 | 33.8 | 56.1% | 83 |
| 10_VitaSalute | 204 | 46.0 | 65.2% | 136 | 33.0 | 55.1% | 340 |
| 11_FIDMAG | 58 | 41.9 | 58.6% | 52 | 39.5 | 53.8% | 110 |
| 12_Creteil | 34 | 36.1 | 38.2% | 52 | 34.9 | 55.8% | 86 |
| 13_UCLA | 238 | 46.5 | 52.9% | 156 | 43.2 | 49.4% | 394 |
| 15_Mannheim | 41 | 40.8 | 56.1% | 37 | 40.7 | 54.1% | 78 |
| 16_UCSD | 39 | 45.8 | 71.8% | 58 | 48.8 | 48.3% | 97 |
| 17_Grenoble | 18 | 43.3 | 50.0% | 12 | 43.6 | 66.7% | 30 |
| 19_SaoPaulo | 40 | 29.1 | 65.0% | 42 | 27.4 | 35.7% | 82 |
| 20_Pittsburgh | 77 | 33.5 | 74.0% | 36 | 34.1 | 69.4% | 113 |
| 21_Columbia | 75 | 39.6 | 64.0% | 0 |  |  | 75 |
| 22_Singapour | 44 | 34.5 | 59.1% | 44 | 34.1 | 52.3% | 88 |
| 23_Halifax | 36 | 45.4 | 58.3% | 67 | 33.6 | 62.7% | 103 |
| TOTAL | 1482 | 39.6 | 60.7% | 1551 | 35.1 | 51.1% | 3033 |

## Table S2. Available clinical data for each site

|  | **1_Munster** | | | **2_UNSW** | | | **3.1_UNC** | | | **4_UCT** | | | **6_Cardiff** | | | **7.1_Edimburgh** | | |
| --- | --- | --- | --- | --- | --- | --- | --- | --- | --- | --- | --- | --- | --- | --- | --- | --- | --- | --- |
|  | **count** | **mean** | **std** | **count** | **mean** | **std** | **count** | **mean** | **std** | **count** | **mean** | **std** | **count** | **mean** | **std** | **count** | **mean** | **std** |
| Age of Onset | 41 | 26.0 | 9.6 | 57 | 16.1 | 3.6 | NA |  |  | 20 | 22.0 | 4.4 | 50 | 19.6 | 7.1 | NA |  |  |
| Illness Duration | 41 | 12.9 | 10.1 | 57 | 9.2 | 4.1 | NA |  |  | 20 | 7.9 | 5.6 | 50 | 24.0 | 8.7 | NA |  |  |
| Density of Episodes | 38 | 1.3 | 1.6 | 27 | 2.2 | 1.5 | NA |  |  | NA |  |  | NA |  |  | NA |  |  |
| Depression score |  |  |  |  |  |  |  |  |  |  |  |  |  |  |  |  |  |  |
| HDRS-17 |  |  |  |  |  |  |  |  |  |  |  |  |  |  |  |  |  |  |
| HDRS-21 | 35 | 22.3 | 5.1 |  |  |  |  |  |  | 20 | 2.8 | 4.1 | 63 | 3.8 | 3.6 | 12 | 4.2 | 7.6 |
| MADRS |  |  |  | 48 | 10.6 | 9.7 | 57 | 15.6 | 11.7 |  |  |  |  |  |  |  |  |  |
| Number of Depressive Episodes | 41 | 6.5 | 4.9 | 55 | 9.4 | 7.1 | 57 | 12.5 | 8.0 | NA |  |  | NA |  |  | NA |  |  |
| Mania Score (YMRS) | 40 | 4.0 | 4.4 | 48 | 4.9 | 4.4 | 57 | 4.9 | 5.0 | 20 | 1.7 | 3.9 | 63 | 2.7 | 2.8 | 11 | 3.5 | 4.1 |
| Number of Manic Episodes | 40 | 2.4 | 3.1 | 27 | 9.4 | 7.2 | 56 | 9.9 | 8.9 | NA |  |  | NA |  |  | NA |  |  |
| Total Episodes | 40 | 9.0 | 6.2 | 27 | 18.6 | 11.1 | 56 | 22.2 | 14.2 | NA |  |  | NA |  |  | NA |  |  |
| On Medication | 41 | 0.9 | 0.3 | 58 | 0.8 | 0.4 | 55 | 0.3 | 0.5 | 20 | 1.0 | 0.0 | 63 | 0.8 | 0.4 | 11 | 0.5 | 0.5 |
| Antipsychotics | 41 | 0.7 | 0.5 | 58 | 0.3 | 0.5 | 55 | 0.1 | 0.3 | 20 | 0.7 | 0.5 | 63 | 0.4 | 0.5 | 11 | 0.4 | 0.5 |
| Anticonvulsants | 41 | 0.3 | 0.5 | 55 | 0.6 | 0.5 | 55 | 0.2 | 0.4 | 20 | 0.6 | 0.5 | 63 | 0.4 | 0.5 | NA |  |  |
| Antidepressants | 41 | 0.5 | 0.5 | 58 | 0.4 | 0.5 | 55 | 0.2 | 0.4 | 20 | 0.1 | 0.2 | 63 | 0.5 | 0.5 | 11 | 0.2 | 0.4 |
| Lithium | 41 | 0.3 | 0.5 | 58 | 0.3 | 0.4 | 55 | 0.1 | 0.3 | 19 | 0.4 | 0.5 | 63 | 0.2 | 0.4 | 11 | 0.2 | 0.4 |
| Lifetime alcohol abuse | NA |  |  | 2 | 1.0 | 0.0 | NA |  |  | NA |  |  | NA |  |  | NA |  |  |
| History of psychotic symptoms | NA |  |  | 56 | 0.3 | 0.5 | 57 | 0.8 | 0.4 | 20 | 1.0 | 0.0 | 63 | 0.2 | 0.4 | NA |  |  |
| Onset time |  |  |  |  |  |  | NA |  |  |  |  |  |  |  |  | NA |  |  |
| Early | 12 |  |  | 46 |  |  |  |  |  | 4 |  |  | 28 |  |  |  |  |  |
| Intermediate | 21 |  |  | 11 |  |  |  |  |  | 16 |  |  | 20 |  |  |  |  |  |
| Late | 8 |  |  |  |  |  |  |  |  |  |  |  | 2 |  |  |  |  |  |
| BD Type |  |  |  |  |  |  |  |  |  |  |  |  | NA |  |  |  |  |  |
| BD1 | 27 |  |  | 32 |  |  | 45 |  |  | 20 |  |  |  |  |  | 12 |  |  |
| BD2 | 13 |  |  | 26 |  |  | 12 |  |  |  |  |  |  |  |  |  |  |  |
| Mood phase |  |  |  |  |  |  |  |  |  |  |  |  |  |  |  | NA |  |  |
| Depressed | 41 |  |  | 2 |  |  | 28 |  |  |  |  |  |  |  |  |  |  |  |
| Euthymic |  |  |  |  |  |  | 20 |  |  | 20 |  |  | 63 |  |  |  |  |  |
| Hypomanic |  |  |  |  |  |  | 2 |  |  |  |  |  |  |  |  |  |  |  |
| Manic |  |  |  |  |  |  | 2 |  |  |  |  |  |  |  |  |  |  |  |

NA: Not Available

## Table S2. Available clinical data for each site (continuation)

|  | **7.2_Edimburgh** | | | **9.2_Oslo_Malt** | | | **10_VitaSalute** | | | **11_FIDMAG** | | | **12_Creteil** | | | **13_UCLA** | | |
| --- | --- | --- | --- | --- | --- | --- | --- | --- | --- | --- | --- | --- | --- | --- | --- | --- | --- | --- |
|  | **count** | **mean** | **std** | **count** | **mean** | **std** | **count** | **mean** | **std** | **count** | **mean** | **std** | **count** | **mean** | **std** | **count** | **mean** | **std** |
| Age of Onset | NA |  |  | 41 | 16.0 | 6.0 | 204 | 30.8 | 10.4 | 56 | 26.1 | 8.8 | 30 | 22.2 | 8.0 | 224 | 29.6 | 10.5 |
| Illness Duration | NA |  |  | 41 | 20.6 | 6.8 | 204 | 15.2 | 10.4 | 56 | 15.6 | 8.7 | 30 | 12.7 | 9.7 | 224 | 16.4 | 11.0 |
| Density of Episodes | NA |  |  | NA |  |  | 198 | 0.8 | 0.9 | 44 | 0.6 | 0.5 | NA |  |  | NA |  |  |
| Depression score |  |  |  |  |  |  | NA |  |  |  |  |  |  |  |  | NA |  |  |
| HDRS-17 |  |  |  |  |  |  |  |  |  |  |  |  |  |  |  |  |  |  |
| HDRS-21 | 27 | 6.3 | 4.5 |  |  |  |  |  |  | 54 | 2.3 | 2.8 |  |  |  |  |  |  |
| MADRS |  |  |  | 40 | 9.8 | 7.1 |  |  |  |  |  |  | 30 | 5.3 | 7.4 |  |  |  |
| Number of Depressive Episodes | NA |  |  | NA |  |  | 200 | 5.0 | 4.8 | 46 | 3.5 | 3.7 | NA |  |  | NA |  |  |
| Mania Score (YMRS) | 27 | 2.1 | 2.9 | 38 | 2.6 | 2.4 | NA |  |  | 55 | 1.1 | 1.8 | 29 | 4.5 | 6.0 | NA |  |  |
| Number of Manic Episodes | NA |  |  | NA |  |  | 201 | 3.1 | 3.9 | 48 | 3.1 | 2.7 | NA |  |  | NA |  |  |
| Total Episodes | NA |  |  | NA |  |  | 200 | 8.1 | 7.8 | 45 | 6.7 | 4.7 | NA |  |  | NA |  |  |
| On Medication | 25 | 1.0 | 0.2 | 40 | 0.8 | 0.4 | NA |  |  | 55 | 1.0 | 0.1 | 31 | 1.0 | 0.2 | 230 | 0.9 | 0.3 |
| Antipsychotics | 25 | 0.5 | 0.5 | 39 | 0.2 | 0.4 | NA |  |  | 55 | 0.6 | 0.5 | 31 | 0.5 | 0.5 | 230 | 0.4 | 0.5 |
| Anticonvulsants | 25 | 0.4 | 0.5 | 40 | 0.6 | 0.5 | NA |  |  | 54 | 0.4 | 0.5 | 31 | 0.5 | 0.5 | 230 | 0.2 | 0.4 |
| Antidepressants | 25 | 0.4 | 0.5 | 40 | 0.3 | 0.5 | NA |  |  | 54 | 0.3 | 0.5 | 31 | 0.3 | 0.5 | 230 | 0.2 | 0.4 |
| Lithium | 25 | 0.6 | 0.5 | 40 | 0.1 | 0.2 | NA |  |  | 54 | 0.8 | 0.4 | 31 | 0.5 | 0.5 | 230 | 0.7 | 0.5 |
| Lifetime alcohol abuse | NA |  |  | NA |  |  | NA |  |  | NA |  |  | NA |  |  | NA |  |  |
| History of psychotic symptoms | NA |  |  | NA |  |  | 204 | 0.3 | 0.4 | 54 | 0.8 | 0.4 | 24 | 0.9 | 0.3 | 223 | 0.8 | 0.4 |
| Onset time | NA |  |  |  |  |  |  |  |  |  |  |  |  |  |  |  |  |  |
| Early |  |  |  | 32 |  |  | 27 |  |  | 11 |  |  | 9 |  |  | 32 |  |  |
| Intermediate |  |  |  | 8 |  |  | 116 |  |  | 34 |  |  | 19 |  |  | 131 |  |  |
| Late |  |  |  | 1 |  |  | 61 |  |  | 11 |  |  | 2 |  |  | 61 |  |  |
| BD type |  |  |  |  |  |  |  |  |  |  |  |  | NA |  |  | NA |  |  |
| BD1 | 27 |  |  |  |  |  |  |  |  | 58 |  |  |  |  |  |  |  |  |
| BD2 |  |  |  | 42 |  |  |  |  |  |  |  |  |  |  |  |  |  |  |
| Mood phase | NA |  |  |  |  |  |  |  |  |  |  |  |  |  |  | NA |  |  |
| Depressed |  |  |  |  |  |  | 178 |  |  |  |  |  | 11 |  |  |  |  |  |
| Euthymic |  |  |  |  |  |  | 6 |  |  | 57 |  |  | 19 |  |  |  |  |  |
| Hypomanic |  |  |  |  |  |  |  |  |  |  |  |  |  |  |  |  |  |  |
| Manic |  |  |  |  |  |  | 16 |  |  |  |  |  |  |  |  |  |  |  |

NA: Not Available

## Table S2. Available clinical data for each site (continuation)

|  | **15_Mannheim** | | | **16_UCSD** | | | **17_Grenoble** | | | **19_SaoPaulo** | | | **20_Pittsburgh** | | | **21_Columbia** | | | **22_Singapour** | | |
| --- | --- | --- | --- | --- | --- | --- | --- | --- | --- | --- | --- | --- | --- | --- | --- | --- | --- | --- | --- | --- | --- |
|  | **count** | **mean** | **std** | **count** | **mean** | **std** | **count** | **mean** | **std** | **count** | **mean** | **std** | **count** | **mean** | **std** | **count** | **mean** | **std** | **count** | **mean** | **std** |
| Age of Onset | 41 | 23.5 | 10.3 | 39 | 18.0 | 4.0 | 13 | 29.3 | 9.6 | 35 | 23.6 | 6.9 | 77 | 18.3 | 6.1 | 74 | 22.3 | 9.3 | 44 | 30.0 | 10.5 |
| Illness Duration | 41 | 17.2 | 10.9 | 39 | 27.7 | 9.7 | 13 | 14.8 | 8.5 | 35 | 5.3 | 6.3 | 77 | 15.2 | 8.0 | 74 | 18.1 | 11.0 | 43 | 4.6 | 5.3 |
| Density of Episodes | NA |  |  | 39 | 0.5 | 0.3 | 8 | 1.1 | 1.1 | NA |  |  | NA |  |  | 60 | 0.5 | 0.6 | NA |  |  |
| Depression score |  |  |  |  |  |  |  |  |  | NA |  |  |  |  |  |  |  |  | NA |  |  |
| HDRS-17 |  |  |  | 39 | 2.6 | 2.0 |  |  |  |  |  |  | 76 | 11.7 | 8.6 |  |  |  |  |  |  |
| HDRS-21 |  |  |  |  |  |  |  |  |  |  |  |  |  |  |  | 74 | 5.5 | 7.6 |  |  |  |
| MADRS | 41 | 1.9 | 2.9 |  |  |  | 14 | 10.4 | 7.9 |  |  |  |  |  |  |  |  |  |  |  |  |
| Number of Depressive Episodes | NA |  |  | 39 | 8.8 | 7.0 | 12 | 5.4 | 5.5 | NA |  |  | 77 | 2.9 | 1.8 | 60 | 2.3 | 3.7 | NA |  |  |
| Mania Score (YMRS) | NA |  |  | 39 | 1.3 | 1.5 | NA |  |  | NA |  |  | NA |  |  | 74 | 0.8 | 2.6 | 44 | 3.5 | 4.9 |
| Number of Manic Episodes | NA |  |  | 39 | 4.8 | 5.4 | 9 | 4.4 | 3.4 | NA |  |  | NA |  |  | 65 | 3.0 | 3.1 | NA |  |  |
| Total Episodes | NA |  |  | 39 | 13.6 | 10.2 | 9 | 10.7 | 9.1 | NA |  |  | NA |  |  | 60 | 5.4 | 5.9 | NA |  |  |
| On Medication | 41 | 0.8 | 0.4 | 39 | 1.0 | 0.0 | NA |  |  | NA |  |  | 77 | 0.7 | 0.4 | 74 | 0.9 | 0.3 | 44 | 1.0 | 0.0 |
| Antipsychotics | 0 |  |  | 39 | 0.5 | 0.5 | NA |  |  | NA |  |  | 77 | 0.6 | 0.5 | 74 | 0.6 | 0.5 | 44 | 0.8 | 0.4 |
| Anticonvulsants | 41 | 0.6 | 0.5 | 39 | 0.5 | 0.5 | NA |  |  | NA |  |  | NA |  |  | 74 | 0.6 | 0.5 | 44 | 0.5 | 0.5 |
| Antidepressants | 41 | 0.5 | 0.5 | 39 | 0.5 | 0.5 | NA |  |  | NA |  |  | 77 | 0.5 | 0.5 | 74 | 0.1 | 0.3 | 44 | 0.1 | 0.3 |
| Lithium | 41 | 0.3 | 0.5 | 38 | 0.2 | 0.4 | NA |  |  | NA |  |  | NA |  |  | 74 | 0.4 | 0.5 | 44 | 0.4 | 0.5 |
| Lifetime alcohol abuse | NA |  |  | 34 | 0.1 | 0.3 | NA |  |  | NA |  |  | NA |  |  | 74 | 0.2 | 0.4 | 44 | 0.0 | 0.0 |
| History of psychotic  symptoms | NA |  |  | 37 | 0.7 | 0.5 | 14 | 0.3 | 0.5 | 38 | 0.2 | 0.4 | NA |  |  | 74 | 0.7 | 0.5 | 44 | 0.3 | 0.4 |
| Onset time |  |  |  |  |  |  |  |  |  |  |  |  |  |  |  |  |  |  |  |  |  |
| Early | 16 |  |  | 23 |  |  | 1 |  |  | 8 |  |  | 51 |  |  | 31 |  |  | 3 |  |  |
| Intermediate | 20 |  |  | 16 |  |  | 8 |  |  | 26 |  |  | 26 |  |  | 34 |  |  | 28 |  |  |
| Late | 5 |  |  |  |  |  | 4 |  |  | 1 |  |  |  |  |  | 9 |  |  | 13 |  |  |
| BD type | NA |  |  |  |  |  |  |  |  |  |  |  |  |  |  |  |  |  |  |  |  |
| BD1 |  |  |  | 39 | 1.0 | 0.0 | 6 |  |  | 17 |  |  | 77 |  |  | 67 |  |  | 44 |  |  |
| BD2 |  |  |  |  |  |  | 8 |  |  | 21 |  |  |  |  |  | 7 |  |  |  |  |  |
| Mood phase | NA |  |  |  |  |  |  |  |  | NA |  |  |  |  |  |  |  |  | NA |  |  |
| Depressed |  |  |  |  |  |  |  |  |  |  |  |  | 33 |  |  | 20 |  |  |  |  |  |
| Euthymic |  |  |  | 39 |  |  | 15 |  |  |  |  |  | 44 |  |  | 53 |  |  |  |  |  |
| Hypomanic |  |  |  |  |  |  |  |  |  |  |  |  |  |  |  |  |  |  |  |  |  |
| Manic |  |  |  |  |  |  |  |  |  |  |  |  |  |  |  |  |  |  |  |  |  |

**Table S3. Demographic details of the original samples (resampled sites)**

|  | **Bipolar Patients** | | | **Healthy Controls** | | |  | **Age** | | **Sex** | |
| --- | --- | --- | --- | --- | --- | --- | --- | --- | --- | --- | --- |
|  | N | Age | %F | N | Age | %F | N Tot. | ***t*** | ***p*-value** | **χ^2^** | ***p*-value** |
| *1 Munster* |  |  |  |  |  |  |  |  |  |  |  |
| Original sample | 41 | 39.0 | 51.2% | 432 | 37.4 | 56.5% | 473 | -0.81 | 0.42 | 0.23 | 0.63 |
| Final sample |  |  |  | **122** | **37.9** | **53.3%** | 163 | -0.49 | 0.62 | 0.00 | 0.96 |
| Original vs. Final (HC) |  |  |  |  |  |  |  | -0.41 | 0.68 | 0.28 | 0.60 |
| *5 KI* |  |  |  |  |  |  |  |  |  |  |  |
| Original sample | 77 | 41.7 | 62.3% | 12 | 42.5 | 25.0% | 89 | 0.25 | 0.80 | **4.49** | **0.03** |
| Final sample | **38** | **41.3** | **65.8%** |  |  |  | 50 | 0.39 | 0.70 | **4.61** | **0.03** |
| Original vs. Final (BD) |  |  |  |  |  |  |  | 0.25 | 0.80 | 0.02 | 0.88 |
| *8.1 IoL* |  |  |  |  |  |  |  |  |  |  |  |
| Original sample | 35 | 35.0 | 51.4% | 161 | 33.0 | 46.0% | 196 | -1.16 | 0.25 | 0.16 | 0.69 |
| Final sample |  |  |  | **104** | **33.1** | **40.4%** | 139 | -0.88 | 0.38 | 0.89 | 0.34 |
| Original vs. Final (HC) |  |  |  |  |  |  |  | -0.40 | 0.69 | 0.59 | 0.44 |
| *8.2 IoL* |  |  |  |  |  |  |  |  |  |  |  |
| Original sample | 18 | 35.9 | 72.2% | 217 | 26.4 | 48.8% | 235 | **-3.45** | **<0.001** | 2.76 | 0.10 |
| Final sample |  |  |  | **57** | **25.0** | **49.1%** | 75 | **-3.55** | **<0.001** | 2.09 | 0.15 |
| Original vs. Final (HC) |  |  |  |  |  |  |  | 0.83 | 0.41 | 0.01 | 0.91 |
| *9.1 Oslo_TOP* |  |  |  |  |  |  |  |  |  |  |  |
| Original sample | 57 | 31.5 | 50.9% | 275 | 31.9 | 41.5% | 332 | 0.36 | 0.72 | 1.35 | 0.25 |
| Final sample |  |  |  | **174** | **32.4** | **41.4%** | 231 | 0.63 | 0.52 | 1.21 | 0.27 |
| Original vs. Final (HC) |  |  |  |  |  |  |  | -0.63 | 0.53 | 0.01 | 0.93 |

## Table S4. Diffusion tensor imaging acquisitions parameters for each site

| **Cohort** | **Scanner** | **Field Strength** | **Nb of Acquisitions** | **Voxel size (mm)** | **Slice Thickness** | **Nb of gradient directions** | **b-values** | **Nb of b=0 scans** | **Reference** |
| --- | --- | --- | --- | --- | --- | --- | --- | --- | --- |
| 1_Munster | Phillips Intera | 3T | 1 | 1.8 × 1.8 | 3.6 | 20 | 1000 | 1 | Repple et al., 2017 |
| 2_UNSW | Philips Achieva | 3T | 2 |  | 2.5 | 32 | 1000 | 1 | Roberts et al., 2016 |
| 3.1_UNC | Siemens Allegra | 3T | 1 | 2 × 2 | 2 | 30 | 1000 | 2 | Bauer et al., 2015 |
| 3.2_UNC | Phillips Intera | 3T | 1 | 2.3 × 2.3 | 2.3 | 35 | 900 | 1 | Benedetti et al., 2011 |
| 4_UCT | Siemens Allegra | 3T | 1 | 1.8 × 1.8 | 4 | 30 | 1000 | 2 | ~ |
| 5_KI | GE Signa Excite | 1.5T | 1 | 0.9375 × 0.9375 | 2.5 | 45 | 1000 | 6 |  |
| 6_Cardiff | GE HD | 3T | 2 | 2.4 × 2.4 | 2.4 | 30 | 1200 | 3 | Caseras et al., 2015 |
| 7.1_Edimburgh | GE Signa | 1.5T | 1 | 2.5 × 2.5 | 2.5 | 65 | 1000 | 7 | Sprooten et al., 2011. 2013 |
| 7.2_Edimburgh | Siemens Magnetom Verio | 3T | 1 | 2.5 x 2.5 | 2.5 | 56 | 1000 | 6 | Alloza et al., 2016. 2017 |
| 8.1_IoL | Siemens | 3T | 1 | 1.56 × 1.56 | 3 | 12 | 1000 | 1 | ~ |
| 8.2_IoL | Siemens Magnetom Allegra | 3T | 1 | 1.7 × 1.7 | 3 | 32 | 1000 | 1 | ~ |
| 8.3_IoL | Siemens Magnetom Allegra | 3T | 1 | 1.7 × 1.7 | 3 | 55 | 800 | 1 | ~ |
| 9.1_Oslo_TOP | GE HDxt | 3T | 1 | 1.875 × 1.875 | 2.5 mm | 30 | 1000 | 2 | Tønnesen et al., in press |
| 9.2_Oslo_Malt | Philips Achieva | 3T | 1 | 2 × 2 | 2 mm | 32 | 1000 | 2 | Elvsåshagen et al., 2015 |
| 10_VitaSalute | Phillips Intera | 3T | 1 | 2.14 × 3.71 | 2.3 | 35 | 900 | 1 | Bolttini et al., 2015; Polletti et al.. 2015 |
| 11_FIDMAG | GE Signa | 3T | 1 | 2.2 × 2.2 | 3 | 55 | 1500 | 1 | Canales-Rodriguez et al., 2013 |
| 12_Creteil | Siemens TrioTrim | 3T | 1 | 2 × 2 | 2 | 41 | 1000 | 1 | Sarrazin et al., 2014 |
| 13_UCLA | Phillips Achieva | 3T | 2 | 2 × 2 | 2 | 30 | 1000 | 5 | Abramovic et al., 2018 |
| 15_Mannheim | Siemens TrioTrim | 3T | 1 | 2 × 2 | 2 | 41 | 1000 | 1 | Sarrazin et al., 2014 |
| 16_UCSD | GE Signa | 3T | 1 | 2.5 × 2.5 | 2.5 | 51 | 1000 | 2 | McKenna et al., 2015 |
| 17_Grenoble | Philips Achieva | 3T | 1 | 3 × 3 | 3 | 30 | 1400 | 5 | Favre et al., 2016 |
| 19_SaoPaulo | Siemens Espree | 1.5T | 2 | 2 × 2 | 2.7 | 64 | 1000 | 1 | Serpa et al., 2017 |
| 20_Pittsburgh | Siemens TrioTrim | 3T | 1 | 2 × 2 | 2 | 41 | 1000 | 1 | Sarrazin et al., 2014 |
| 21_Columbia | Phillips Ingenia | 3T | 1 | 1.75 × 1.75 | 2 | 16 | 1000 | 1 | ~ |
| 22_Singapour | Philips Achieva | 3T | 1 | 1 × 1 | 3 | 15 | 800 | 1 | Chan et al., 2010 |
| 23_Halifax | GE Signa | 1.5T | 2 | 1.875 × 1.875 | 2 | 31 | 900 | 2 | ~ |

## Table S5. Description of the ROIs

| **Abbreviation** | **Full tract name** |
| --- | --- |
| AverageFA | Full skeleton average FA |
| ACR (L and R) | Anterior *corona radiata* |
| ALIC (L and R) | Anterior limb of internal capsule |
| BCC | Body of *corpus callosum* |
| CC (BCC. GCC and SCC) | Corpus callosum |
| CGC (L and R) | Cingulum (cingulate gyrus) |
| CGH (Land R) | Cingulum (hippocampal portion) |
| CR (L and R) | *Corona radiata* |
| CST (L and R) | Corticospinal tract |
| EC (L and R) | External capsule |
| FX | *Fornix* |
| FXST (L and R) | *Fornix* (cres) / *Stria terminalis* |
| GCC | *Genu* of *corpus callosum* |
| IC (L and R) | Internal capsule |
| IFO (L and R) | Inferior fronto-occipital fasciculus |
| PCR (L and R) | Posterior *corona radiata* |
| PLIC (L and R) | Posterior limb of internal capsule |
| PTR (L and R) | Posterior thalamic radiation |
| RLIC (L and R) | Retrolenticular part of internal capsule |
| SCC | *Splenium* of *corpus callosum* |
| SCR (L and R) | Superior *corona radiata* |
| SFO (L and R) | Superior fronto-occipital fasciculus |
| SLF (L and R) | Superior longitudinal fasciculus |
| SS (L and R) | Sagittal *stratum* |
| UNC (L and R) | *Uncinate* fasciculus |

## Table S6. Mega-analysis: Linear mixed model results for FA differences between bipolar patients and healthy controls after controlling for average FA. age and sex

| **ROI** | ***β*** | ***s.e*** | ***t*-value** | **P_corr_>\|*t*\|** | **R^2^** | **[0.025** | **0.975]** | **Sign.** |
| --- | --- | --- | --- | --- | --- | --- | --- | --- |
| *Projection fibers* | |  |  |  |  |  |  |  |
| PTR.R | 0.0070 | 0.0011 | 6.2650 | 1.88E-08 | 0.0129 | 0.0061 | 0.0222 | *** |
| PTR.L | 0.0052 | 0.0011 | 4.6106 | 1.84E-04 | 0.0071 | 0.0023 | 0.0143 | *** |
| ACR.L | 0.0035 | 0.0008 | 4.3049 | 7.59E-04 | 0.0059 | 0.0017 | 0.0127 | *** |
| CR.L | 0.0020 | 0.0006 | 3.4714 | 2.31E-02 | 0.0038 | 0.0007 | 0.0096 | * |
| ACR.R | 0.0027 | 0.0008 | 3.1646 | 6.90E-02 | 0.0033 | 0.0004 | 0.0086 | NS |
| CST.R | -0.0043 | 0.0016 | -2.7357 | 2.76E-01 | 0.0025 | 0.0002 | 0.0074 | NS |
| CST.L | -0.0041 | 0.0016 | -2.5615 | 4.61E-01 | 0.0022 | 0.0001 | 0.0068 | NS |
| PLIC.L | -0.0024 | 0.0009 | -2.6194 | 3.90E-01 | 0.0022 | 0.0001 | 0.0069 | NS |
| CR.R | 0.0014 | 0.0006 | 2.5148 | 5.26E-01 | 0.0020 | 0.0001 | 0.0066 | NS |
| PLIC.R | -0.0021 | 0.0009 | -2.3315 | 8.71E-01 | 0.0018 | 0.0001 | 0.0061 | NS |
| ALIC.L | 0.0016 | 0.0009 | 1.8808 | 1.00E+00 | 0.0012 | 0.0000 | 0.0049 | NS |
| PCR.R | 0.0015 | 0.0008 | 1.8055 | 1.00E+00 | 0.0011 | 0.0000 | 0.0047 | NS |
| ALIC.R | 0.0014 | 0.0008 | 1.6572 | 1.00E+00 | 0.0009 | 0.0000 | 0.0044 | NS |
| IC.R | -0.0010 | 0.0006 | -1.6292 | 1.00E+00 | 0.0009 | 0.0000 | 0.0043 | NS |
| RLIC.R | -0.0015 | 0.0010 | -1.4661 | 1.00E+00 | 0.0007 | 0.0000 | 0.0039 | NS |
| PCR.L | 0.0011 | 0.0008 | 1.3801 | 1.00E+00 | 0.0006 | 0.0000 | 0.0037 | NS |
| IC.L | -0.0007 | 0.0006 | -1.1002 | 1.00E+00 | 0.0004 | 0.0000 | 0.0031 | NS |
| RLIC.L | -0.0010 | 0.0009 | -1.1352 | 1.00E+00 | 0.0004 | 0.0000 | 0.0032 | NS |
| SCR.L | 0.0007 | 0.0008 | 0.8565 | 1.00E+00 | 0.0002 | 0.0000 | 0.0027 | NS |
| SCR.R | 0.0002 | 0.0008 | 0.2327 | 1.00E+00 | 0.0000 | 0.0000 | 0.0018 | NS |
| *Association fibers* | |  |  |  |  |  |  |  |
| CGC.R | 0.0102 | 0.0012 | 8.7512 | 0.00E+00 | 0.0244 | 0.0147 | 0.0365 | *** |
| CGC.L | 0.0101 | 0.0012 | 8.5796 | 0.00E+00 | 0.0235 | 0.0139 | 0.0353 | *** |
| EC.L | 0.0035 | 0.0007 | 4.6869 | 1.28E-04 | 0.0072 | 0.0024 | 0.0145 | *** |
| UNC.L | 0.0077 | 0.0019 | 4.1426 | 1.55E-03 | 0.0057 | 0.0016 | 0.0124 | ** |
| UNC.R | 0.0072 | 0.0017 | 4.1581 | 1.45E-03 | 0.0057 | 0.0016 | 0.0124 | ** |
| EC.R | 0.0028 | 0.0007 | 4.0297 | 2.52E-03 | 0.0054 | 0.0014 | 0.0119 | ** |
| SLF.L | 0.0027 | 0.0007 | 3.8338 | 5.67E-03 | 0.0048 | 0.0011 | 0.0110 | ** |
| IFO.R | 0.0052 | 0.0016 | 3.3555 | 3.53E-02 | 0.0038 | 0.0006 | 0.0094 | * |
| SFO.R | 0.0031 | 0.0012 | 2.6982 | 3.08E-01 | 0.0024 | 0.0002 | 0.0072 | NS |
| SFO.L | 0.0028 | 0.0012 | 2.3277 | 8.80E-01 | 0.0018 | 0.0001 | 0.0062 | NS |
| IFO.L | 0.0034 | 0.0017 | 2.0041 | 1.00E+00 | 0.0014 | 0.0000 | 0.0053 | NS |
| CGH.L | -0.0027 | 0.0016 | -1.7013 | 1.00E+00 | 0.0010 | 0.0000 | 0.0045 | NS |
| FX.ST.R | 0.0021 | 0.0013 | 1.6852 | 1.00E+00 | 0.0009 | 0.0000 | 0.0045 | NS |
| FX.ST.L | 0.0014 | 0.0012 | 1.1819 | 1.00E+00 | 0.0005 | 0.0000 | 0.0033 | NS |
| SS.R | 0.0012 | 0.0010 | 1.1667 | 1.00E+00 | 0.0005 | 0.0000 | 0.0033 | NS |
| CGH.R | 0.0004 | 0.0017 | 0.2501 | 1.00E+00 | 0.0000 | 0.0000 | 0.0018 | NS |
| *Commissural fibers* | |  |  |  |  |  |  |  |
| CC | 0.0093 | 0.0007 | 13.2778 | 0.00E+00 | 0.0535 | 0.0390 | 0.0701 | *** |
| BCC | 0.0115 | 0.0011 | 10.7620 | 0.00E+00 | 0.0363 | 0.0243 | 0.0506 | *** |
| GCC | 0.0091 | 0.0009 | 10.2340 | 0.00E+00 | 0.0327 | 0.0213 | 0.0463 | *** |
| SCC | 0.0056 | 0.0008 | 7.1338 | 5.39E-11 | 0.0164 | 0.0086 | 0.0266 | *** |
| FX | 0.0153 | 0.0023 | 6.5437 | 3.10E-09 | 0.0145 | 0.0072 | 0.0243 | *** |

*p_corr_<0.05; **p_corr_<0.01; ***p_corr_<0.001; ns: not significant

**Table S7.** **Mega-analysis:** **Linear mixed model results for the interaction between diagnosis and age after controlling for sex**

| **ROI** | ***β*** | ***s.e*** | ***t*-value** | **P_corr_>\|*t*\|** | **R^2^** | **[0.025** | **0.975]** | **Sign.** |
| --- | --- | --- | --- | --- | --- | --- | --- | --- |
| *Projection fibers* | |  |  |  |  |  |  |  |
| SCR.L | -0.0003 | 0.0001 | -3.5478 | 1.74E-02 | 0.0041 | 0.0008 | 0.0099 | * |
| SCR.R | -0.0003 | 0.0001 | -3.5516 | 1.71E-02 | 0.004 | 0.0008 | 0.0099 | * |
| PLIC.L | -0.0003 | 0.0001 | -3.4784 | 2.25E-02 | 0.0039 | 0.0007 | 0.0096 | * |
| RLIC.R | -0.0003 | 0.0001 | -2.9505 | 1.41E-01 | 0.0028 | 0.0003 | 0.0079 | NS |
| IC.L | -0.0002 | 0.0001 | -2.6094 | 4.01E-01 | 0.0021 | 0.0001 | 0.0067 | NS |
| PLIC.R | -0.0002 | 0.0001 | -2.41 | 7.05E-01 | 0.0019 | 0.0001 | 0.0062 | NS |
| RLIC.L | -0.0002 | 0.0001 | -2.4163 | 6.93E-01 | 0.0019 | 0.0001 | 0.0063 | NS |
| IC.R | -0.0002 | 0.0001 | -2.3367 | 8.59E-01 | 0.0017 | 0.0000 | 0.006 | NS |
| CR.L | -0.0001 | 0.0001 | -2.0933 | 1.00E+00 | 0.0014 | 0.0000 | 0.0054 | NS |
| CR.R | -0.0001 | 0.0001 | -1.5521 | 1.00E+00 | 0.0008 | 0.0000 | 0.0041 | NS |
| CST.L | -0.0002 | 0.0001 | -1.5389 | 1.00E+00 | 0.0008 | 0.0000 | 0.004 | NS |
| PTR.R | -0.0002 | 0.0001 | -1.5621 | 1.00E+00 | 0.0008 | 0.0000 | 0.0041 | NS |
| PCR.L | -0.0001 | 0.0001 | -1.275 | 1.00E+00 | 0.0005 | 0.0000 | 0.0035 | NS |
| PCR.R | -0.0001 | 0.0001 | -1.1434 | 1.00E+00 | 0.0004 | 0.0000 | 0.0032 | NS |
| CST.R | -0.0001 | 0.0001 | -1.0153 | 1.00E+00 | 0.0003 | 0.0000 | 0.0029 | NS |
| ACR.L | 0.0000 | 0.0001 | -0.4703 | 1.00E+00 | 0.0001 | 0.0000 | 0.002 | NS |
| ACR.R | 0.0000 | 0.0001 | 0.5016 | 1.00E+00 | 0.0001 | 0.0000 | 0.0021 | NS |
| PTR.L | 0.0000 | 0.0001 | -0.4327 | 1.00E+00 | 0.0001 | 0.0000 | 0.002 | NS |
| ALIC.L | 0.0000 | 0.0001 | 0.044 | 1.00E+00 | 0.0000 | 0.0000 | 0.0017 | NS |
| ALIC.R | 0.0000 | 0.0001 | -0.0098 | 1.00E+00 | 0.0000 | 0.0000 | 0.0017 | NS |
| *Association fibers* | |  |  |  |  |  |  |  |
| CGC.L | 0.0004 | 0.0001 | 3.552 | 1.71E-02 | 0.004 | 0.0008 | 0.0099 | * |
| CGC.R | 0.0004 | 0.0001 | 3.1548 | 7.14E-02 | 0.0032 | 0.0004 | 0.0085 | NS |
| IFO.R | 0.0003 | 0.0001 | 2.1526 | 1.00E+00 | 0.0015 | 0.0000 | 0.0056 | NS |
| EC.L | 0.0001 | 0.0001 | 1.9514 | 1.00E+00 | 0.0012 | 0.0000 | 0.005 | NS |
| CGH.L | 0.0002 | 0.0001 | 1.4147 | 1.00E+00 | 0.0006 | 0.0000 | 0.0038 | NS |
| EC.R | 0.0001 | 0.0001 | 1.269 | 1.00E+00 | 0.0005 | 0.0000 | 0.0034 | NS |
| UNC.L | 0.0002 | 0.0002 | 1.0521 | 1.00E+00 | 0.0004 | 0.0000 | 0.003 | NS |
| SS.R | -0.0001 | 0.0001 | -0.9555 | 1.00E+00 | 0.0003 | 0.0000 | 0.0028 | NS |
| UNC.R | 0.0001 | 0.0002 | 0.9697 | 1.00E+00 | 0.0003 | 0.0000 | 0.0029 | NS |
| CGH.R | 0.0001 | 0.0002 | 0.7894 | 1.00E+00 | 0.0002 | 0.0000 | 0.0025 | NS |
| IFO.L | 0.0001 | 0.0001 | 0.6747 | 1.00E+00 | 0.0001 | 0.0000 | 0.0023 | NS |
| SS.L | 0.0000 | 0.0001 | 0.3963 | 1.00E+00 | 0.0001 | 0.0000 | 0.0019 | NS |
| FX.ST.L | 0.0000 | 0.0001 | 0.0921 | 1.00E+00 | 0.0000 | 0.0000 | 0.0017 | NS |
| FX.ST.R | 0.0000 | 0.0001 | 0.3056 | 1.00E+00 | 0.0000 | 0.0000 | 0.0018 | NS |
| SFO.L | 0.0000 | 0.0001 | -0.3409 | 1.00E+00 | 0.0000 | 0.0000 | 0.0019 | NS |
| SFO.R | 0.0000 | 0.0001 | -0.1596 | 1.00E+00 | 0.0000 | 0.0000 | 0.0017 | NS |
| *Commissural fibers* | |  |  |  |  |  |  |  |
| FX | 0.0006 | 0.0002 | 3.0578 | 9.90E-02 | 0.0031 | 0.0004 | 0.0083 | NS |
| BCC | 0.0003 | 0.0001 | 2.8544 | 1.91E-01 | 0.0026 | 0.0002 | 0.0076 | NS |
| CC | 0.0002 | 0.0001 | 2.7143 | 2.94E-01 | 0.0023 | 0.0002 | 0.0071 | NS |
| SCC | 0.0002 | 0.0001 | 2.2962 | 9.56E-01 | 0.0016 | 0.0000 | 0.0058 | NS |
| GCC | 0.0001 | 0.0001 | 1.0939 | 1.00E+00 | 0.0004 | 0.0000 | 0.0031 | NS |
| *AverageFA* | 0.0000 | 0.0001 | 0.2674 | 1.00E+00 | 0.0000 | 0.0000 | 0.0018 | NS |

*p_corr_<0.05; **p_corr_<0.01; ***p_corr_<0.001; ns: not significant

**Table S8.** **Mega-analysis:** **Linear mixed model results for the interaction between diagnosis and sex after controlling for age**

| **ROI** | ***β*** | ***s.e*** | ***t*-value** | **P_corr_>\|*t*\|** | **R^2^** | **[0.025** | **0.975]** | **Sign.** |
| --- | --- | --- | --- | --- | --- | --- | --- | --- |
| *Projection fibers* | |  |  |  |  |  |  |  |
| CST.L | 0.0036 | 0.0032 | 1.1213 | 1.00E+00 | 4.00E-04 | 0.0000 | 0.0031 | NS |
| PLIC.L | 0.0018 | 0.0019 | 0.9093 | 1.00E+00 | 3.00E-04 | 0.0000 | 0.0027 | NS |
| RLIC.R | -0.0022 | 0.0023 | -0.947 | 1.00E+00 | 3.00E-04 | 0.0000 | 0.0028 | NS |
| ALIC.L | 0.0016 | 0.002 | 0.778 | 1.00E+00 | 2.00E-04 | 0.0000 | 0.0025 | NS |
| PLIC.R | 0.0017 | 0.0019 | 0.8925 | 1.00E+00 | 2.00E-04 | 0.0000 | 0.0027 | NS |
| PTR.L | -0.0022 | 0.0026 | -0.8601 | 1.00E+00 | 2.00E-04 | 0.0000 | 0.0026 | NS |
| RLIC.L | -0.0018 | 0.0021 | -0.8785 | 1.00E+00 | 2.00E-04 | 0.0000 | 0.0027 | NS |
| ALIC.R | 0.0012 | 0.002 | 0.5892 | 1.00E+00 | 1.00E-04 | 0.0000 | 0.0022 | NS |
| CR.R | -7.00E-04 | 0.0016 | -0.4065 | 1.00E+00 | 1.00E-04 | 0.0000 | 0.0019 | NS |
| CST.R | -0.0019 | 0.0031 | -0.6116 | 1.00E+00 | 1.00E-04 | 0.0000 | 0.0022 | NS |
| IC.L | 8.00E-04 | 0.0015 | 0.5028 | 1.00E+00 | 1.00E-04 | 0.0000 | 0.0021 | NS |
| IC.R | 8.00E-04 | 0.0016 | 0.4977 | 1.00E+00 | 1.00E-04 | 0.0000 | 0.002 | NS |
| PCR.L | -0.0012 | 0.002 | -0.6023 | 1.00E+00 | 1.00E-04 | 0.0000 | 0.0022 | NS |
| SCR.L | -0.0011 | 0.0019 | -0.5508 | 1.00E+00 | 1.00E-04 | 0.0000 | 0.0021 | NS |
| ACR.L | 3.00E-04 | 0.0021 | 0.1473 | 1.00E+00 | 0.0000 | 0.0000 | 0.0017 | NS |
| ACR.R | -8.00E-04 | 0.0022 | -0.3583 | 1.00E+00 | 0.0000 | 0.0000 | 0.0019 | NS |
| CR.L | -6.00E-04 | 0.0017 | -0.3605 | 1.00E+00 | 0.0000 | 0.0000 | 0.0019 | NS |
| PCR.R | -5.00E-04 | 0.002 | -0.2282 | 1.00E+00 | 0.0000 | 0.0000 | 0.0018 | NS |
| PTR.R | 4.00E-04 | 0.0025 | 0.1701 | 1.00E+00 | 0.0000 | 0.0000 | 0.0017 | NS |
| SCR.R | -6.00E-04 | 0.0019 | -0.3349 | 1.00E+00 | 0.0000 | 0.0000 | 0.0019 | NS |
| *Association fibers* | |  |  |  |  |  |  |  |
| IFO.R | -0.01 | 0.0033 | -3.0479 | 1.02E-01 | 0.0029 | 0.0000 | 0.008 | NS |
| CGC.R | 0.005 | 0.0028 | 1.7798 | 1.00E+00 | 0.001 | 0.0000 | 0.0045 | NS |
| CGH.L | -0.0059 | 0.0034 | -1.7031 | 1.00E+00 | 9.00E-04 | 0.0000 | 0.0043 | NS |
| CGC.L | 0.0045 | 0.0029 | 1.5653 | 1.00E+00 | 8.00E-04 | 0.0000 | 0.004 | NS |
| CGH.R | -0.0037 | 0.0036 | -1.0018 | 1.00E+00 | 3.00E-04 | 0.0000 | 0.0029 | NS |
| IFO.L | -0.0036 | 0.0035 | -1.0194 | 1.00E+00 | 3.00E-04 | 0.0000 | 0.0029 | NS |
| SFO.L | 0.0025 | 0.0027 | 0.957 | 1.00E+00 | 3.00E-04 | 0.0000 | 0.0028 | NS |
| FX.ST.L | -0.0016 | 0.0025 | -0.6307 | 1.00E+00 | 1.00E-04 | 0.0000 | 0.0022 | NS |
| SS.L | 0.0014 | 0.0022 | 0.626 | 1.00E+00 | 1.00E-04 | 0.0000 | 0.0022 | NS |
| SS.R | -0.0012 | 0.0024 | -0.5259 | 1.00E+00 | 1.00E-04 | 0.0000 | 0.0021 | NS |
| EC.L | 4.00E-04 | 0.0018 | 0.2114 | 1.00E+00 | 0.0000 | 0.0000 | 0.0018 | NS |
| EC.R | -3.00E-04 | 0.0017 | -0.194 | 1.00E+00 | 0.0000 | 0.0000 | 0.0018 | NS |
| FX.ST.R | -5.00E-04 | 0.0027 | -0.1923 | 1.00E+00 | 0.0000 | 0.0000 | 0.0018 | NS |
| SFO.R | 5.00E-04 | 0.0026 | 0.1898 | 1.00E+00 | 0.0000 | 0.0000 | 0.0018 | NS |
| UNC.L | -4.00E-04 | 0.0038 | -0.0933 | 1.00E+00 | 0.0000 | 0.0000 | 0.0017 | NS |
| UNC.R | -9.00E-04 | 0.0037 | -0.2371 | 1.00E+00 | 0.0000 | 0.0000 | 0.0018 | NS |
| *Commissural fibers* | |  |  |  |  |  |  |  |
| BCC | 0.003 | 0.0027 | 1.1148 | 1.00E+00 | 4.00E-04 | 0.0000 | 0.0031 | NS |
| CC | 0.002 | 0.002 | 1.0086 | 1.00E+00 | 3.00E-04 | 0.0000 | 0.0029 | NS |
| SCC | 0.0014 | 0.0018 | 0.7638 | 1.00E+00 | 2.00E-04 | 0.0000 | 0.0024 | NS |
| FX | 0.002 | 0.0048 | 0.4108 | 1.00E+00 | 1.00E-04 | 0.0000 | 0.002 | NS |
| GCC | 0.0011 | 0.0023 | 0.5008 | 1.00E+00 | 1.00E-04 | 0.0000 | 0.0021 | NS |
| *AverageFA* | 8.00E-04 | 0.0012 | 0.6545 | 1.00E+00 | 1.00E-04 | 0.0000 | 0.0023 | NS |

*p_corr_<0.05; **p_corr_<0.01; ***p_corr_<0.001; ns: not significant

**Table S9.** **Mega-analysis:** **Linear mixed model results for the main effect of the age of onset after controlling for age and sex**

| **ROI** | ***β*** | ***s.e*** | ***t*-value** | **P_corr_>\|*t*\|** | **R^2^** | **[0.025** | **0.975]** | **Sign.** |
| --- | --- | --- | --- | --- | --- | --- | --- | --- |
| *Projection fibers* | |  |  |  |  |  |  |  |
| ACR.L | 0.0003 | 0.0001 | 2.6615 | 3.48E-01 | 0.0071 | 0.0006 | 0.0207 | NS |
| CR.L | 0.0002 | 0.0001 | 2.3380 | 8.61E-01 | 0.0055 | 0.0002 | 0.0179 | NS |
| CR.R | 0.0002 | 0.0001 | 2.2438 | 1.00E+00 | 0.0050 | 0.0001 | 0.0171 | NS |
| ACR.R | 0.0002 | 0.0001 | 1.9447 | 1.00E+00 | 0.0038 | 0.0000 | 0.0149 | NS |
| SCR.R | 0.0002 | 0.0001 | 1.7881 | 1.00E+00 | 0.0033 | 0.0000 | 0.0137 | NS |
| PCR.R | 0.0002 | 0.0001 | 1.6102 | 1.00E+00 | 0.0026 | 0.0000 | 0.0125 | NS |
| PCR.L | 0.0002 | 0.0001 | 1.4896 | 1.00E+00 | 0.0023 | 0.0000 | 0.0117 | NS |
| PTR.L | 0.0002 | 0.0001 | 1.3563 | 1.00E+00 | 0.0019 | 0.0000 | 0.0108 | NS |
| SCR.L | 0.0001 | 0.0001 | 1.3337 | 1.00E+00 | 0.0018 | 0.0000 | 0.0107 | NS |
| ALIC.L | -0.0001 | 0.0001 | -1.0869 | 1.00E+00 | 0.0012 | 0.0000 | 0.0089 | NS |
| CST.R | -0.0002 | 0.0002 | -0.9603 | 1.00E+00 | 0.0009 | 0.0000 | 0.0082 | NS |
| CST.L | -0.0002 | 0.0002 | -0.9041 | 1.00E+00 | 0.0008 | 0.0000 | 0.0079 | NS |
| PTR.R | 0.0001 | 0.0001 | 0.9111 | 1.00E+00 | 0.0008 | 0.0000 | 0.0080 | NS |
| RLIC.L | 0.0001 | 0.0001 | 0.8016 | 1.00E+00 | 0.0007 | 0.0000 | 0.0074 | NS |
| IC.L | 0.0001 | 0.0001 | 0.7028 | 1.00E+00 | 0.0005 | 0.0000 | 0.0068 | NS |
| PLIC.R | 0.0001 | 0.0001 | 0.5126 | 1.00E+00 | 0.0003 | 0.0000 | 0.0060 | NS |
| IC.R | 0.0000 | 0.0001 | 0.4846 | 1.00E+00 | 0.0002 | 0.0000 | 0.0059 | NS |
| RLIC.R | 0.0000 | 0.0001 | -0.2657 | 1.00E+00 | 0.0001 | 0.0000 | 0.0051 | NS |
| ALIC.R | 0.0000 | 0.0001 | 0.2089 | 1.00E+00 | 0.0000 | 0.0000 | 0.0050 | NS |
| PLIC.L | 0.0000 | 0.0001 | 0.1223 | 1.00E+00 | 0.0000 | 0.0000 | 0.0049 | NS |
| *Association fibers* | |  |  |  |  |  |  |  |
| IFO.R | 0.0006 | 0.0002 | 3.2871 | 4.60E-02 | 0.0110 | 0.0020 | 0.0270 | * |
| EC.R | 0.0003 | 0.0001 | 2.9818 | 1.29E-01 | 0.0088 | 0.0011 | 0.0236 | NS |
| CGC.L | 0.0005 | 0.0002 | 2.8530 | 1.94E-01 | 0.0080 | 0.0008 | 0.0222 | NS |
| IFO.L | 0.0005 | 0.0002 | 2.6649 | 3.44E-01 | 0.0074 | 0.0007 | 0.0212 | NS |
| UNC.L | 0.0005 | 0.0002 | 2.2933 | 9.69E-01 | 0.0054 | 0.0002 | 0.0179 | NS |
| SFO.R | 0.0003 | 0.0001 | 2.2371 | 1.00E+00 | 0.0051 | 0.0002 | 0.0172 | NS |
| SS.L | 0.0002 | 0.0001 | 1.9842 | 1.00E+00 | 0.0040 | 0.0001 | 0.0152 | NS |
| UNC.R | 0.0004 | 0.0002 | 1.9647 | 1.00E+00 | 0.0040 | 0.0001 | 0.0152 | NS |
| EC.L | 0.0002 | 0.0001 | 1.9242 | 1.00E+00 | 0.0037 | 0.0000 | 0.0146 | NS |
| SS.R | 0.0002 | 0.0001 | 1.7892 | 1.00E+00 | 0.0033 | 0.0000 | 0.0138 | NS |
| CGC.R | 0.0003 | 0.0002 | 1.7141 | 1.00E+00 | 0.0029 | 0.0000 | 0.0130 | NS |
| FX.ST.R | 0.0001 | 0.0002 | 0.9169 | 1.00E+00 | 0.0008 | 0.0000 | 0.0080 | NS |
| CGH.L | 0.0001 | 0.0002 | 0.6215 | 1.00E+00 | 0.0004 | 0.0000 | 0.0065 | NS |
| CGH.R | 0.0001 | 0.0002 | 0.3677 | 1.00E+00 | 0.0001 | 0.0000 | 0.0054 | NS |
| FX.ST.L | 0.0000 | 0.0001 | 0.1687 | 1.00E+00 | 0.0000 | 0.0000 | 0.0049 | NS |
| SFO.L | 0.0000 | 0.0001 | 0.1087 | 1.00E+00 | 0.0000 | 0.0000 | 0.0049 | NS |
| *Commissural fibers* | |  |  |  |  |  |  |  |
| BCC | 0.0003 | 0.0002 | 1.8871 | 1.00E+00 | 0.0035 | 0.0000 | 0.0143 | NS |
| CC | 0.0002 | 0.0001 | 1.3904 | 1.00E+00 | 0.0019 | 0.0000 | 0.0107 | NS |
| FX | 0.0003 | 0.0003 | 0.8971 | 1.00E+00 | 0.0009 | 0.0000 | 0.0081 | NS |
| GCC | 0.0001 | 0.0001 | 0.9023 | 1.00E+00 | 0.0008 | 0.0000 | 0.0078 | NS |
| SCC | 0.0000 | 0.0001 | 0.2070 | 1.00E+00 | 0.0000 | 0.0000 | 0.0050 | NS |
| *AverageFA* | 0.0001 | 0.0001 | 0.9947 | 1.00E+00 | 0.0009 | 0.0000 | 0.0083 | NS |

*p_corr_<0.05; **p_corr_<0.01; ***p_corr_<0.001; ns: not significant

**Table S10.** **Mega-analysis:** **Linear mixed model results for the main effect of illness duration after controlling for age and sex**

| **ROI** | ***β*** | ***s.e*** | ***t*-value** | **P_corr_>\|*t*\|** | **R^2^** | **[0.025** | **0.975]** | **Sign.** |
| --- | --- | --- | --- | --- | --- | --- | --- | --- |
| *Projection fibers* | |  |  |  |  |  |  |  |
| ACR.L | -0.0003 | 0.0001 | -2.7453 | 2.71E-01 | 0.0076 | 0.0007 | 0.0215 | NS |
| CR.L | -0.0002 | 0.0001 | -2.5332 | 5.04E-01 | 0.0064 | 0.0004 | 0.0196 | NS |
| CR.R | -0.0002 | 0.0001 | -2.1679 | 1.00E+00 | 0.0047 | 0.0001 | 0.0165 | NS |
| ACR.R | -0.0002 | 0.0001 | -1.8110 | 1.00E+00 | 0.0033 | 0.0000 | 0.0139 | NS |
| SCR.R | -0.0002 | 0.0001 | -1.7912 | 1.00E+00 | 0.0033 | 0.0000 | 0.0138 | NS |
| PCR.L | -0.0002 | 0.0001 | -1.7643 | 1.00E+00 | 0.0032 | 0.0000 | 0.0136 | NS |
| PTR.L | -0.0002 | 0.0001 | -1.6710 | 1.00E+00 | 0.0029 | 0.0000 | 0.0129 | NS |
| PCR.R | -0.0002 | 0.0001 | -1.5641 | 1.00E+00 | 0.0025 | 0.0000 | 0.0121 | NS |
| SCR.L | -0.0002 | 0.0001 | -1.4233 | 1.00E+00 | 0.0021 | 0.0000 | 0.0113 | NS |
| PTR.R | -0.0002 | 0.0001 | -1.2733 | 1.00E+00 | 0.0017 | 0.0000 | 0.0102 | NS |
| RLIC.L | -0.0001 | 0.0001 | -1.0650 | 1.00E+00 | 0.0012 | 0.0000 | 0.0089 | NS |
| ALIC.L | 0.0001 | 0.0001 | 1.0584 | 1.00E+00 | 0.0011 | 0.0000 | 0.0088 | NS |
| CST.R | 0.0002 | 0.0002 | 1.0531 | 1.00E+00 | 0.0011 | 0.0000 | 0.0087 | NS |
| PLIC.L | -0.0001 | 0.0001 | -0.8603 | 1.00E+00 | 0.0008 | 0.0000 | 0.0077 | NS |
| PLIC.R | -0.0001 | 0.0001 | -0.8208 | 1.00E+00 | 0.0007 | 0.0000 | 0.0075 | NS |
| CST.L | 0.0001 | 0.0002 | 0.6888 | 1.00E+00 | 0.0005 | 0.0000 | 0.0068 | NS |
| IC.L | -0.0001 | 0.0001 | -0.6047 | 1.00E+00 | 0.0004 | 0.0000 | 0.0064 | NS |
| IC.R | 0.0000 | 0.0001 | -0.4595 | 1.00E+00 | 0.0002 | 0.0000 | 0.0058 | NS |
| RLIC.R | 0.0001 | 0.0001 | 0.4572 | 1.00E+00 | 0.0002 | 0.0000 | 0.0058 | NS |
| ALIC.R | 0.0000 | 0.0001 | -0.2475 | 1.00E+00 | 0.0001 | 0.0000 | 0.0051 | NS |
| *Association fibers* | |  |  |  |  |  |  |  |
| CGC.L | -0.0005 | 0.0002 | -3.4074 | 3.00E-02 | 0.0114 | 0.0022 | 0.0275 | * |
| IFO.R | -0.0006 | 0.0002 | -3.1400 | 7.64E-02 | 0.0100 | 0.0016 | 0.0255 | NS |
| IFO.L | -0.0005 | 0.0002 | -2.7288 | 2.84E-01 | 0.0077 | 0.0008 | 0.0218 | NS |
| EC.R | -0.0002 | 0.0001 | -2.5777 | 4.44E-01 | 0.0066 | 0.0004 | 0.0199 | NS |
| EC.L | -0.0002 | 0.0001 | -2.2117 | 1.00E+00 | 0.0049 | 0.0001 | 0.0169 | NS |
| UNC.L | -0.0004 | 0.0002 | -2.1851 | 1.00E+00 | 0.0049 | 0.0001 | 0.0170 | NS |
| SFO.R | -0.0003 | 0.0001 | -2.0946 | 1.00E+00 | 0.0045 | 0.0001 | 0.0161 | NS |
| SS.L | -0.0002 | 0.0001 | -2.0805 | 1.00E+00 | 0.0044 | 0.0001 | 0.0160 | NS |
| CGC.R | -0.0003 | 0.0002 | -1.9837 | 1.00E+00 | 0.0039 | 0.0001 | 0.0150 | NS |
| SS.R | -0.0003 | 0.0001 | -1.9477 | 1.00E+00 | 0.0039 | 0.0001 | 0.0150 | NS |
| UNC.R | -0.0004 | 0.0002 | -1.8732 | 1.00E+00 | 0.0036 | 0.0000 | 0.0145 | NS |
| FX.ST.R | -0.0001 | 0.0002 | -0.8743 | 1.00E+00 | 0.0008 | 0.0000 | 0.0078 | NS |
| CGH.L | -0.0001 | 0.0002 | -0.6632 | 1.00E+00 | 0.0004 | 0.0000 | 0.0067 | NS |
| SFO.L | -0.0001 | 0.0001 | -0.5378 | 1.00E+00 | 0.0003 | 0.0000 | 0.0061 | NS |
| CGH.R | -0.0001 | 0.0002 | -0.3894 | 1.00E+00 | 0.0001 | 0.0000 | 0.0055 | NS |
| FX.ST.L | 0.0000 | 0.0001 | -0.2810 | 1.00E+00 | 0.0001 | 0.0000 | 0.0052 | NS |
| *Commissural fibers* | |  |  |  |  |  |  |  |
| BCC | -0.0004 | 0.0002 | -2.5197 | 5.23E-01 | 0.0063 | 0.0004 | 0.0194 | NS |
| CC | -0.0002 | 0.0001 | -2.1649 | 1.00E+00 | 0.0045 | 0.0001 | 0.0162 | NS |
| FX | -0.0004 | 0.0003 | -1.3161 | 1.00E+00 | 0.0018 | 0.0000 | 0.0107 | NS |
| SCC | -0.0001 | 0.0001 | -1.3216 | 1.00E+00 | 0.0017 | 0.0000 | 0.0103 | NS |
| GCC | -0.0002 | 0.0001 | -1.2453 | 1.00E+00 | 0.0015 | 0.0000 | 0.0098 | NS |
| *AverageFA* | -0.0001 | 0.0001 | -1.2889 | 1.00E+00 | 0.0016 | 0.0000 | 0.0100 | NS |

*p_corr_<0.05; **p_corr_<0.01; ***p_corr_<0.001; ns: not significant

**Table S11.** **Mega-analysis:** **Linear mixed model results for the main effect of antipsychotic treatment after controlling for age and sex**

| **ROI** | ***β*** | ***s.e*** | ***t*-value** | **P_corr_>\|*t*\|** | **R^2^** | **[0.025** | **0.975]** | **Sign.** |
| --- | --- | --- | --- | --- | --- | --- | --- | --- |
| *Projection fibers* | |  |  |  |  |  |  |  |
| PTR.R | -0.0072 | 0.0023 | -3.0870 | 9.19E-02 | 0.0107 | 0.0014 | 0.0286 | NS |
| ALIC.R | -0.0044 | 0.0020 | -2.2611 | 1.00E+00 | 0.0058 | 0.0001 | 0.0201 | NS |
| PTR.L | -0.0051 | 0.0024 | -2.1038 | 1.00E+00 | 0.0050 | 0.0001 | 0.0188 | NS |
| ACR.R | -0.0036 | 0.0021 | -1.7238 | 1.00E+00 | 0.0034 | 0.0000 | 0.0155 | NS |
| SCR.R | 0.0025 | 0.0018 | 1.3796 | 1.00E+00 | 0.0022 | 0.0000 | 0.0129 | NS |
| CST.R | 0.0038 | 0.0030 | 1.2665 | 1.00E+00 | 0.0018 | 0.0000 | 0.0119 | NS |
| ACR.L | -0.0020 | 0.0020 | -1.0048 | 1.00E+00 | 0.0012 | 0.0000 | 0.0101 | NS |
| RLIC.R | 0.0023 | 0.0022 | 1.0322 | 1.00E+00 | 0.0012 | 0.0000 | 0.0103 | NS |
| PLIC.R | 0.0016 | 0.0020 | 0.8259 | 1.00E+00 | 0.0008 | 0.0000 | 0.0090 | NS |
| RLIC.L | -0.0017 | 0.0021 | -0.8433 | 1.00E+00 | 0.0008 | 0.0000 | 0.0091 | NS |
| ALIC.L | -0.0013 | 0.0019 | -0.6673 | 1.00E+00 | 0.0005 | 0.0000 | 0.0080 | NS |
| CR.L | -0.0006 | 0.0016 | -0.3817 | 1.00E+00 | 0.0002 | 0.0000 | 0.0066 | NS |
| CR.R | -0.0007 | 0.0016 | -0.4303 | 1.00E+00 | 0.0002 | 0.0000 | 0.0068 | NS |
| IC.L | -0.0003 | 0.0015 | -0.2197 | 1.00E+00 | 0.0001 | 0.0000 | 0.0061 | NS |
| PCR.R | -0.0006 | 0.0019 | -0.2868 | 1.00E+00 | 0.0001 | 0.0000 | 0.0063 | NS |
| PLIC.L | 0.0006 | 0.0020 | 0.3111 | 1.00E+00 | 0.0001 | 0.0000 | 0.0064 | NS |
| SCR.L | 0.0005 | 0.0019 | 0.2494 | 1.00E+00 | 0.0001 | 0.0000 | 0.0062 | NS |
| CST.L | 0.0002 | 0.0032 | 0.0465 | 1.00E+00 | 0.0000 | 0.0000 | 0.0058 | NS |
| IC.R | -0.0002 | 0.0015 | -0.1418 | 1.00E+00 | 0.0000 | 0.0000 | 0.0059 | NS |
| PCR.L | 0.0004 | 0.0019 | 0.2056 | 1.00E+00 | 0.0000 | 0.0000 | 0.0061 | NS |
| *Association fibers* | |  |  |  |  |  |  |  |
| CGC.R | -0.0063 | 0.0027 | -2.3384 | 8.62E-01 | 0.0062 | 0.0002 | 0.0208 | NS |
| CGH.R | 0.0064 | 0.0034 | 1.8703 | 1.00E+00 | 0.0039 | 0.0000 | 0.0165 | NS |
| SS.L | -0.0036 | 0.0022 | -1.6379 | 1.00E+00 | 0.0031 | 0.0000 | 0.0148 | NS |
| FX.ST.L | -0.0038 | 0.0026 | -1.4904 | 1.00E+00 | 0.0025 | 0.0000 | 0.0136 | NS |
| IFO.R | 0.0041 | 0.0032 | 1.2832 | 1.00E+00 | 0.0019 | 0.0000 | 0.0121 | NS |
| UNC.R | -0.0042 | 0.0035 | -1.2046 | 1.00E+00 | 0.0017 | 0.0000 | 0.0115 | NS |
| CGC.L | -0.0030 | 0.0029 | -1.0630 | 1.00E+00 | 0.0013 | 0.0000 | 0.0104 | NS |
| CGH.L | 0.0036 | 0.0033 | 1.0819 | 1.00E+00 | 0.0013 | 0.0000 | 0.0105 | NS |
| SFO.R | -0.0025 | 0.0025 | -1.0080 | 1.00E+00 | 0.0012 | 0.0000 | 0.0102 | NS |
| IFO.L | 0.0019 | 0.0033 | 0.5738 | 1.00E+00 | 0.0004 | 0.0000 | 0.0075 | NS |
| FX.ST.R | -0.0013 | 0.0026 | -0.4725 | 1.00E+00 | 0.0003 | 0.0000 | 0.0070 | NS |
| SFO.L | -0.0008 | 0.0026 | -0.3147 | 1.00E+00 | 0.0001 | 0.0000 | 0.0064 | NS |
| SS.R | 0.0006 | 0.0023 | 0.2544 | 1.00E+00 | 0.0001 | 0.0000 | 0.0062 | NS |
| UNC.L | 0.0012 | 0.0036 | 0.3209 | 1.00E+00 | 0.0001 | 0.0000 | 0.0064 | NS |
| EC.L | -0.0003 | 0.0017 | -0.1763 | 1.00E+00 | 0.0000 | 0.0000 | 0.0060 | NS |
| EC.R | 0.0003 | 0.0017 | 0.1942 | 1.00E+00 | 0.0000 | 0.0000 | 0.0060 | NS |
| *Commissural fibers* | |  |  |  |  |  |  |  |
| GCC | -0.0075 | 0.0023 | -3.2668 | 4.98E-02 | 0.0117 | 0.0018 | 0.0302 | * |
| CC | -0.0063 | 0.0021 | -3.0509 | 1.04E-01 | 0.0103 | 0.0012 | 0.0279 | NS |
| BCC | -0.0071 | 0.0028 | -2.5688 | 4.56E-01 | 0.0075 | 0.0004 | 0.0231 | NS |
| SCC | -0.0040 | 0.0019 | -2.1257 | 1.00E+00 | 0.0050 | 0.0001 | 0.0188 | NS |
| FX | -0.0084 | 0.0054 | -1.5540 | 1.00E+00 | 0.0029 | 0.0000 | 0.0143 | NS |
| *AverageFA* | -0.0010 | 0.0011 | -0.8975 | 1.00E+00 | 0.0009 | 0.0000 | 0.0092 | NS |

*p_corr_<0.05; **p_corr_<0.01; ***p_corr_<0.001; ns: not significant

**Table S12.** **Mega-analysis:** **Linear mixed model results for the main effect of anticonvulsant treatment after controlling for age and sex**

| **ROI** | ***β*** | ***s.e*** | ***t*-value** | **P_corr_>\|*t*\|** | **R^2^** | **[0.025** | **0.975]** | **Sign.** |
| --- | --- | --- | --- | --- | --- | --- | --- | --- |
| *Projection fibers* | |  |  |  |  |  |  |  |
| PCR.R | -0.0069 | 0.0021 | -3.3148 | 4.22E-02 | 0.0136 | 0.0024 | 0.0338 | * |
| CR.R | -0.0054 | 0.0017 | -3.2047 | 6.18E-02 | 0.0126 | 0.0019 | 0.0322 | NS |
| PTR.R | -0.0080 | 0.0025 | -3.1994 | 6.30E-02 | 0.0126 | 0.0019 | 0.0322 | NS |
| PTR.L | -0.0077 | 0.0026 | -3.0027 | 1.21E-01 | 0.0111 | 0.0014 | 0.0299 | NS |
| ALIC.R | -0.0059 | 0.0021 | -2.7939 | 2.35E-01 | 0.0095 | 0.0009 | 0.0273 | NS |
| ACR.R | -0.0057 | 0.0022 | -2.6141 | 4.01E-01 | 0.0085 | 0.0006 | 0.0255 | NS |
| CR.L | -0.0041 | 0.0017 | -2.4766 | 5.93E-01 | 0.0076 | 0.0004 | 0.0240 | NS |
| ACR.L | -0.0050 | 0.0021 | -2.4018 | 7.28E-01 | 0.0071 | 0.0003 | 0.0232 | NS |
| PCR.L | -0.0048 | 0.0021 | -2.3433 | 8.52E-01 | 0.0069 | 0.0003 | 0.0228 | NS |
| IC.R | -0.0036 | 0.0016 | -2.1823 | 1.00E+00 | 0.0058 | 0.0001 | 0.0208 | NS |
| SCR.R | -0.0041 | 0.0020 | -2.0911 | 1.00E+00 | 0.0055 | 0.0001 | 0.0202 | NS |
| ALIC.L | -0.0037 | 0.0020 | -1.8152 | 1.00E+00 | 0.0040 | 0.0000 | 0.0173 | NS |
| IC.L | -0.0027 | 0.0016 | -1.7522 | 1.00E+00 | 0.0038 | 0.0000 | 0.0168 | NS |
| CST.R | -0.0052 | 0.0032 | -1.6540 | 1.00E+00 | 0.0033 | 0.0000 | 0.0159 | NS |
| SCR.L | -0.0030 | 0.0021 | -1.4442 | 1.00E+00 | 0.0027 | 0.0000 | 0.0144 | NS |
| RLIC.L | -0.0031 | 0.0022 | -1.3928 | 1.00E+00 | 0.0024 | 0.0000 | 0.0139 | NS |
| RLIC.R | -0.0027 | 0.0024 | -1.1045 | 1.00E+00 | 0.0015 | 0.0000 | 0.0116 | NS |
| CST.L | -0.0023 | 0.0034 | -0.6705 | 1.00E+00 | 0.0006 | 0.0000 | 0.0085 | NS |
| PLIC.R | -0.0015 | 0.0021 | -0.6992 | 1.00E+00 | 0.0006 | 0.0000 | 0.0088 | NS |
| PLIC.L | 0.0000 | 0.0022 | 0.0061 | 1.00E+00 | 0.0000 | 0.0000 | 0.0062 | NS |
| *Association fibers* | |  |  |  |  |  |  |  |
| CGC.R | -0.0110 | 0.0029 | -3.8530 | 5.55E-03 | 0.0180 | 0.0044 | 0.0403 | ** |
| CGC.L | -0.0111 | 0.0030 | -3.7420 | 8.61E-03 | 0.0169 | 0.0039 | 0.0388 | ** |
| UNC.L | -0.0097 | 0.0039 | -2.4862 | 5.77E-01 | 0.0078 | 0.0004 | 0.0244 | NS |
| CGH.L | -0.0081 | 0.0034 | -2.3756 | 7.81E-01 | 0.0068 | 0.0002 | 0.0226 | NS |
| EC.R | -0.0041 | 0.0018 | -2.3298 | 8.83E-01 | 0.0066 | 0.0002 | 0.0223 | NS |
| FX.ST.L | -0.0051 | 0.0027 | -1.8931 | 1.00E+00 | 0.0044 | 0.0000 | 0.0182 | NS |
| SS.L | -0.0039 | 0.0023 | -1.6724 | 1.00E+00 | 0.0035 | 0.0000 | 0.0163 | NS |
| SS.R | -0.0041 | 0.0025 | -1.6238 | 1.00E+00 | 0.0033 | 0.0000 | 0.0158 | NS |
| UNC.R | -0.0060 | 0.0038 | -1.5720 | 1.00E+00 | 0.0031 | 0.0000 | 0.0154 | NS |
| FX.ST.R | -0.0035 | 0.0029 | -1.2180 | 1.00E+00 | 0.0018 | 0.0000 | 0.0124 | NS |
| IFO.L | -0.0035 | 0.0035 | -1.0100 | 1.00E+00 | 0.0013 | 0.0000 | 0.0109 | NS |
| EC.L | -0.0016 | 0.0018 | -0.9167 | 1.00E+00 | 0.0010 | 0.0000 | 0.0102 | NS |
| SFO.L | 0.0021 | 0.0028 | 0.7723 | 1.00E+00 | 0.0008 | 0.0000 | 0.0092 | NS |
| SFO.R | -0.0018 | 0.0027 | -0.6788 | 1.00E+00 | 0.0006 | 0.0000 | 0.0086 | NS |
| IFO.R | -0.0013 | 0.0034 | -0.3913 | 1.00E+00 | 0.0002 | 0.0000 | 0.0071 | NS |
| CGH.R | -0.0010 | 0.0037 | -0.2715 | 1.00E+00 | 0.0001 | 0.0000 | 0.0066 | NS |
| *Commissural fibers* | |  |  |  |  |  |  |  |
| BCC | -0.0158 | 0.0029 | -5.4986 | 2.27E-06 | 0.0361 | 0.0152 | 0.0649 | *** |
| CC | -0.0110 | 0.0022 | -5.0988 | 1.88E-05 | 0.0307 | 0.0117 | 0.0579 | *** |
| FX | -0.0270 | 0.0058 | -4.6796 | 1.48E-04 | 0.0275 | 0.0097 | 0.0536 | *** |
| GCC | -0.0109 | 0.0024 | -4.4986 | 3.46E-04 | 0.0240 | 0.0077 | 0.0489 | *** |
| SCC | -0.0047 | 0.0020 | -2.3090 | 9.33E-01 | 0.0065 | 0.0002 | 0.0220 | NS |
| *AverageFA* | -0.0044 | 0.0012 | -3.6932 | 1.04E-02 | 0.0159 | 0.0034 | 0.0373 | * |

*p_corr_<0.05; **p_corr_<0.01; ***p_corr_<0.001; ns: not significant

**Table S13.** **Mega-analysis:** **Linear mixed model results for the main effect of lithium treatment after controlling for age and sex**

| **ROI** | ***β*** | ***s.e*** | ***t*-value** | **P_corr_>\|*t*\|** | **R^2^** | **[0.025** | **0.975]** | **Sign.** |
| --- | --- | --- | --- | --- | --- | --- | --- | --- |
| *Projection fibers* | |  |  |  |  |  |  |  |
| ACR.R | 0.0098 | 0.0022 | 4.3826 | 5.84E-04 | 0.0246 | 0.0081 | 0.0495 | *** |
| CR.R | 0.0073 | 0.0017 | 4.2513 | 1.04E-03 | 0.0230 | 0.0072 | 0.0473 | ** |
| ACR.L | 0.0080 | 0.0021 | 3.7595 | 8.03E-03 | 0.0182 | 0.0046 | 0.0404 | ** |
| PTR.R | 0.0091 | 0.0026 | 3.5568 | 1.75E-02 | 0.0162 | 0.0036 | 0.0375 | * |
| RLIC.R | 0.0087 | 0.0025 | 3.5297 | 1.93E-02 | 0.0162 | 0.0036 | 0.0375 | * |
| IC.L | 0.0056 | 0.0016 | 3.5275 | 1.95E-02 | 0.0158 | 0.0034 | 0.0369 | * |
| CR.L | 0.0059 | 0.0017 | 3.4685 | 2.42E-02 | 0.0155 | 0.0033 | 0.0365 | * |
| IC.R | 0.0059 | 0.0017 | 3.4902 | 2.24E-02 | 0.0155 | 0.0032 | 0.0364 | * |
| RLIC.L | 0.0068 | 0.0022 | 3.0481 | 1.05E-01 | 0.0121 | 0.0018 | 0.0313 | NS |
| SCR.R | 0.0057 | 0.0020 | 2.8866 | 1.76E-01 | 0.0110 | 0.0014 | 0.0294 | NS |
| ALIC.L | 0.0060 | 0.0021 | 2.9003 | 1.68E-01 | 0.0107 | 0.0013 | 0.0290 | NS |
| PTR.L | 0.0075 | 0.0027 | 2.8255 | 2.13E-01 | 0.0103 | 0.0012 | 0.0284 | NS |
| SCR.L | 0.0057 | 0.0021 | 2.7274 | 2.87E-01 | 0.0100 | 0.0010 | 0.0278 | NS |
| PCR.R | 0.0057 | 0.0021 | 2.6698 | 3.41E-01 | 0.0093 | 0.0008 | 0.0268 | NS |
| ALIC.R | 0.0049 | 0.0022 | 2.2939 | 9.70E-01 | 0.0067 | 0.0002 | 0.0223 | NS |
| PLIC.R | 0.0035 | 0.0022 | 1.6092 | 1.00E+00 | 0.0034 | 0.0000 | 0.0159 | NS |
| PCR.L | 0.0030 | 0.0021 | 1.4321 | 1.00E+00 | 0.0027 | 0.0000 | 0.0144 | NS |
| PLIC.L | 0.0029 | 0.0022 | 1.3210 | 1.00E+00 | 0.0023 | 0.0000 | 0.0135 | NS |
| CST.L | 0.0044 | 0.0035 | 1.2713 | 1.00E+00 | 0.0021 | 0.0000 | 0.0129 | NS |
| CST.R | 0.0032 | 0.0032 | 1.0049 | 1.00E+00 | 0.0013 | 0.0000 | 0.0108 | NS |
| *Association fibers* | |  |  |  |  |  |  |  |
| FX.ST.L | 0.0119 | 0.0027 | 4.3822 | 5.85E-04 | 0.0245 | 0.0080 | 0.0493 | *** |
| SS.R | 0.0094 | 0.0026 | 3.6641 | 1.16E-02 | 0.0174 | 0.0042 | 0.0393 | * |
| CGH.L | 0.0111 | 0.0035 | 3.2283 | 5.70E-02 | 0.0131 | 0.0022 | 0.0328 | NS |
| SS.L | 0.0073 | 0.0024 | 3.1023 | 8.74E-02 | 0.0126 | 0.0020 | 0.0320 | NS |
| CGH.R | 0.0116 | 0.0037 | 3.1033 | 8.71E-02 | 0.0121 | 0.0018 | 0.0313 | NS |
| FX.ST.R | 0.0081 | 0.0029 | 2.7559 | 2.63E-01 | 0.0097 | 0.0010 | 0.0275 | NS |
| EC.R | 0.0036 | 0.0018 | 1.9808 | 1.00E+00 | 0.0050 | 0.0001 | 0.0192 | NS |
| EC.L | 0.0036 | 0.0018 | 1.9405 | 1.00E+00 | 0.0049 | 0.0001 | 0.0188 | NS |
| CGC.L | 0.0052 | 0.0031 | 1.6898 | 1.00E+00 | 0.0037 | 0.0000 | 0.0164 | NS |
| UNC.R | 0.0057 | 0.0039 | 1.4547 | 1.00E+00 | 0.0028 | 0.0000 | 0.0146 | NS |
| CGC.R | 0.0034 | 0.0029 | 1.1563 | 1.00E+00 | 0.0017 | 0.0000 | 0.0120 | NS |
| SFO.R | 0.0031 | 0.0027 | 1.1439 | 1.00E+00 | 0.0017 | 0.0000 | 0.0120 | NS |
| UNC.L | 0.0044 | 0.0040 | 1.1083 | 1.00E+00 | 0.0016 | 0.0000 | 0.0118 | NS |
| SFO.L | 0.0028 | 0.0028 | 0.9873 | 1.00E+00 | 0.0013 | 0.0000 | 0.0108 | NS |
| IFO.L | 0.0025 | 0.0036 | 0.6903 | 1.00E+00 | 0.0006 | 0.0000 | 0.0087 | NS |
| IFO.R | 0.0022 | 0.0035 | 0.6514 | 1.00E+00 | 0.0006 | 0.0000 | 0.0085 | NS |
| *Commissural fibers* | |  |  |  |  |  |  |  |
| GCC | 0.0106 | 0.0025 | 4.2885 | 8.87E-04 | 0.0228 | 0.0071 | 0.0470 | *** |
| CC | 0.0063 | 0.0022 | 2.8365 | 2.06E-01 | 0.0102 | 0.0011 | 0.0281 | NS |
| FX | 0.0138 | 0.0059 | 2.3171 | 9.13E-01 | 0.0072 | 0.0003 | 0.0232 | NS |
| BCC | 0.0066 | 0.0030 | 2.1916 | 1.00E+00 | 0.0062 | 0.0002 | 0.0213 | NS |
| SCC | 0.0028 | 0.0021 | 1.3687 | 1.00E+00 | 0.0024 | 0.0000 | 0.0136 | NS |
| *AverageFA* | 0.0054 | 0.0012 | 4.4518 | 4.27E-04 | 0.0239 | 0.0077 | 0.0485 | *** |

*p_corr_<0.05; **p_corr_<0.01; ***p_corr_<0.001; ns: not significant

**Table S14.** **Mega-analysis:** **Linear mixed model results for the main effect of antidepressant treatment after controlling for age and sex**

| **ROI** | ***β*** | ***s.e*** | ***t*-value** | **P_corr_>\|*t*\|** | **R^2^** | **[0.025** | **0.975]** | **Sign.** |
| --- | --- | --- | --- | --- | --- | --- | --- | --- |
| *Projection fibers* | |  |  |  |  |  |  |  |
| ALIC.R | -0.0051 | 0.0020 | -2.5249 | 5.17E-01 | 0.0066 | 0.0003 | 0.0212 | NS |
| SCR.R | -0.0044 | 0.0019 | -2.3167 | 9.13E-01 | 0.0057 | 0.0002 | 0.0196 | NS |
| ACR.L | -0.0042 | 0.0020 | -2.0573 | 1.00E+00 | 0.0045 | 0.0001 | 0.0172 | NS |
| CR.R | -0.0034 | 0.0017 | -2.0765 | 1.00E+00 | 0.0045 | 0.0001 | 0.0174 | NS |
| ACR.R | -0.0041 | 0.0022 | -1.8867 | 1.00E+00 | 0.0038 | 0.0000 | 0.0159 | NS |
| CR.L | -0.0027 | 0.0016 | -1.6719 | 1.00E+00 | 0.0030 | 0.0000 | 0.0142 | NS |
| CST.R | 0.0054 | 0.0031 | 1.7092 | 1.00E+00 | 0.0030 | 0.0000 | 0.0143 | NS |
| ALIC.L | -0.0023 | 0.0020 | -1.1809 | 1.00E+00 | 0.0014 | 0.0000 | 0.0106 | NS |
| SCR.L | -0.0021 | 0.0020 | -1.0522 | 1.00E+00 | 0.0012 | 0.0000 | 0.0099 | NS |
| PLIC.R | -0.0020 | 0.0021 | -0.9514 | 1.00E+00 | 0.0010 | 0.0000 | 0.0093 | NS |
| IC.R | -0.0016 | 0.0016 | -0.9545 | 1.00E+00 | 0.0009 | 0.0000 | 0.0092 | NS |
| PTR.L | -0.0024 | 0.0025 | -0.9436 | 1.00E+00 | 0.0009 | 0.0000 | 0.0092 | NS |
| PCR.L | -0.0013 | 0.0020 | -0.6453 | 1.00E+00 | 0.0004 | 0.0000 | 0.0075 | NS |
| PCR.R | -0.0012 | 0.0020 | -0.6054 | 1.00E+00 | 0.0004 | 0.0000 | 0.0073 | NS |
| PLIC.L | 0.0010 | 0.0021 | 0.4857 | 1.00E+00 | 0.0003 | 0.0000 | 0.0067 | NS |
| RLIC.R | 0.0012 | 0.0023 | 0.5191 | 1.00E+00 | 0.0003 | 0.0000 | 0.0069 | NS |
| IC.L | -0.0007 | 0.0015 | -0.4480 | 1.00E+00 | 0.0002 | 0.0000 | 0.0065 | NS |
| PTR.R | -0.0006 | 0.0024 | -0.2265 | 1.00E+00 | 0.0001 | 0.0000 | 0.0058 | NS |
| RLIC.L | -0.0008 | 0.0022 | -0.3532 | 1.00E+00 | 0.0001 | 0.0000 | 0.0062 | NS |
| CST.L | 0.0002 | 0.0034 | 0.0701 | 1.00E+00 | 0.0000 | 0.0000 | 0.0056 | NS |
| *Association fibers* | |  |  |  |  |  |  |  |
| SFO.R | -0.0068 | 0.0026 | -2.6302 | 3.82E-01 | 0.0074 | 0.0004 | 0.0225 | NS |
| CGC.R | -0.0060 | 0.0028 | -2.1492 | 1.00E+00 | 0.0048 | 0.0001 | 0.0179 | NS |
| UNC.R | -0.0071 | 0.0037 | -1.9309 | 1.00E+00 | 0.0040 | 0.0000 | 0.0163 | NS |
| SS.L | -0.0040 | 0.0023 | -1.7513 | 1.00E+00 | 0.0033 | 0.0000 | 0.0148 | NS |
| EC.L | -0.0028 | 0.0018 | -1.5948 | 1.00E+00 | 0.0027 | 0.0000 | 0.0135 | NS |
| EC.R | -0.0028 | 0.0017 | -1.6061 | 1.00E+00 | 0.0027 | 0.0000 | 0.0136 | NS |
| CGC.L | -0.0042 | 0.0029 | -1.4536 | 1.00E+00 | 0.0022 | 0.0000 | 0.0125 | NS |
| SFO.L | -0.0039 | 0.0027 | -1.4290 | 1.00E+00 | 0.0022 | 0.0000 | 0.0125 | NS |
| IFO.R | -0.0046 | 0.0034 | -1.3635 | 1.00E+00 | 0.0020 | 0.0000 | 0.0120 | NS |
| FX.ST.L | -0.0028 | 0.0027 | -1.0339 | 1.00E+00 | 0.0011 | 0.0000 | 0.0097 | NS |
| IFO.L | -0.0032 | 0.0034 | -0.9335 | 1.00E+00 | 0.0009 | 0.0000 | 0.0092 | NS |
| FX.ST.R | -0.0024 | 0.0028 | -0.8527 | 1.00E+00 | 0.0008 | 0.0000 | 0.0086 | NS |
| UNC.L | -0.0026 | 0.0038 | -0.6753 | 1.00E+00 | 0.0005 | 0.0000 | 0.0077 | NS |
| CGH.R | -0.0020 | 0.0036 | -0.5524 | 1.00E+00 | 0.0003 | 0.0000 | 0.0070 | NS |
| SS.R | 0.0007 | 0.0024 | 0.3065 | 1.00E+00 | 0.0001 | 0.0000 | 0.0060 | NS |
| CGH.L | -0.0005 | 0.0034 | -0.1426 | 1.00E+00 | 0.0000 | 0.0000 | 0.0057 | NS |
| *Commissural fibers* | |  |  |  |  |  |  |  |
| GCC | -0.0051 | 0.0024 | -2.1478 | 1.00E+00 | 0.0047 | 0.0001 | 0.0177 | NS |
| BCC | -0.0056 | 0.0028 | -1.9742 | 1.00E+00 | 0.0041 | 0.0000 | 0.0165 | NS |
| CC | -0.0036 | 0.0021 | -1.6807 | 1.00E+00 | 0.0029 | 0.0000 | 0.0141 | NS |
| FX | -0.0089 | 0.0056 | -1.5776 | 1.00E+00 | 0.0027 | 0.0000 | 0.0137 | NS |
| SCC | 0.0003 | 0.0020 | 0.1396 | 1.00E+00 | 0.0000 | 0.0000 | 0.0057 | NS |
| *AverageFA* | -0.0020 | 0.0012 | -1.6539 | 1.00E+00 | 0.0027 | 0.0000 | 0.0137 | NS |

*p_corr_<0.05; **p_corr_<0.01; ***p_corr_<0.001; ns: not significant

**Table S15.** **Mega-analysis:** **Linear mixed model results for the main effect of the severity of the disease (density of episodes) after controlling for age and sex**

| **ROI** | ***β*** | ***s.e*** | ***t*-value** | **P_corr_>\|*t*\|** | **R^2^** | **[0.025** | **0.975]** | **Sign.** |
| --- | --- | --- | --- | --- | --- | --- | --- | --- |
| *Projection fibers* | |  |  |  |  |  |  |  |
| SCR.R | 0.0036 | 0.0014 | 2.5624 | 4.73E-01 | 0.0145 | 0.0007 | 0.0458 | NS |
| ACR.L | 0.0040 | 0.0016 | 2.5147 | 5.41E-01 | 0.0143 | 0.0006 | 0.0454 | NS |
| CR.R | 0.0032 | 0.0013 | 2.5537 | 4.85E-01 | 0.0143 | 0.0006 | 0.0455 | NS |
| CR.L | 0.0031 | 0.0012 | 2.5493 | 4.91E-01 | 0.0142 | 0.0006 | 0.0453 | NS |
| ACR.R | 0.0036 | 0.0017 | 2.0845 | 1.00E+00 | 0.0100 | 0.0001 | 0.0377 | NS |
| SCR.L | 0.0029 | 0.0015 | 1.9722 | 1.00E+00 | 0.0088 | 0.0001 | 0.0353 | NS |
| ALIC.R | 0.0019 | 0.0014 | 1.3671 | 1.00E+00 | 0.0041 | 0.0000 | 0.0254 | NS |
| PCR.L | 0.0019 | 0.0015 | 1.2839 | 1.00E+00 | 0.0037 | 0.0000 | 0.0244 | NS |
| PCR.R | 0.0018 | 0.0014 | 1.2789 | 1.00E+00 | 0.0037 | 0.0000 | 0.0243 | NS |
| CST.R | 0.0019 | 0.0021 | 0.9099 | 1.00E+00 | 0.0018 | 0.0000 | 0.0192 | NS |
| ALIC.L | 0.0012 | 0.0013 | 0.8765 | 1.00E+00 | 0.0017 | 0.0000 | 0.0187 | NS |
| CST.L | 0.0018 | 0.0021 | 0.8331 | 1.00E+00 | 0.0015 | 0.0000 | 0.0182 | NS |
| RLIC.L | 0.0011 | 0.0015 | 0.7749 | 1.00E+00 | 0.0013 | 0.0000 | 0.0176 | NS |
| PTR.L | 0.0011 | 0.0019 | 0.5846 | 1.00E+00 | 0.0008 | 0.0000 | 0.0155 | NS |
| IC.L | 0.0006 | 0.0011 | 0.5073 | 1.00E+00 | 0.0006 | 0.0000 | 0.0146 | NS |
| IC.R | 0.0006 | 0.0012 | 0.4728 | 1.00E+00 | 0.0005 | 0.0000 | 0.0143 | NS |
| PLIC.L | -0.0004 | 0.0014 | -0.2614 | 1.00E+00 | 0.0002 | 0.0000 | 0.0128 | NS |
| PTR.R | 0.0004 | 0.0019 | 0.2121 | 1.00E+00 | 0.0001 | 0.0000 | 0.0126 | NS |
| PLIC.R | -0.0002 | 0.0014 | -0.1068 | 1.00E+00 | 0.0000 | 0.0000 | 0.0122 | NS |
| RLIC.R | 0.0000 | 0.0016 | 0.0138 | 1.00E+00 | 0.0000 | 0.0000 | 0.0121 | NS |
| *Association fibers* | |  |  |  |  |  |  |  |
| IFO.L | 0.0076 | 0.0025 | 3.0748 | 9.89E-02 | 0.0218 | 0.0028 | 0.0576 | NS |
| FX.ST.L | 0.0047 | 0.0017 | 2.6955 | 3.22E-01 | 0.0160 | 0.0010 | 0.0484 | NS |
| EC.L | 0.0032 | 0.0013 | 2.4325 | 6.79E-01 | 0.0132 | 0.0005 | 0.0435 | NS |
| UNC.R | 0.0060 | 0.0026 | 2.3095 | 9.42E-01 | 0.0122 | 0.0003 | 0.0418 | NS |
| IFO.R | 0.0052 | 0.0023 | 2.2636 | 1.00E+00 | 0.0116 | 0.0003 | 0.0407 | NS |
| UNC.L | 0.0060 | 0.0028 | 2.1621 | 1.00E+00 | 0.0108 | 0.0002 | 0.0393 | NS |
| EC.R | 0.0028 | 0.0013 | 2.1786 | 1.00E+00 | 0.0106 | 0.0002 | 0.0389 | NS |
| SFO.R | 0.0034 | 0.0017 | 2.0770 | 1.00E+00 | 0.0097 | 0.0001 | 0.0371 | NS |
| CGC.R | 0.0040 | 0.0021 | 1.8870 | 1.00E+00 | 0.0079 | 0.0001 | 0.0337 | NS |
| SFO.L | 0.0025 | 0.0017 | 1.4489 | 1.00E+00 | 0.0047 | 0.0000 | 0.0268 | NS |
| CGC.L | 0.0025 | 0.0022 | 1.1777 | 1.00E+00 | 0.0031 | 0.0000 | 0.0228 | NS |
| FX.ST.R | 0.0022 | 0.0020 | 1.1200 | 1.00E+00 | 0.0028 | 0.0000 | 0.0221 | NS |
| SS.L | 0.0013 | 0.0016 | 0.8416 | 1.00E+00 | 0.0016 | 0.0000 | 0.0185 | NS |
| CGH.L | 0.0017 | 0.0025 | 0.7019 | 1.00E+00 | 0.0011 | 0.0000 | 0.0166 | NS |
| CGH.R | -0.0010 | 0.0025 | -0.4058 | 1.00E+00 | 0.0004 | 0.0000 | 0.0138 | NS |
| SS.R | 0.0006 | 0.0018 | 0.3344 | 1.00E+00 | 0.0003 | 0.0000 | 0.0133 | NS |
| *Commissural fibers* | |  |  |  |  |  |  |  |
| FX | 0.0081 | 0.0038 | 2.1252 | 1.00E+00 | 0.0107 | 0.0002 | 0.0391 | NS |
| GCC | 0.0042 | 0.0019 | 2.2101 | 1.00E+00 | 0.0105 | 0.0002 | 0.0386 | NS |
| BCC | 0.0047 | 0.0022 | 2.1405 | 1.00E+00 | 0.0102 | 0.0002 | 0.0381 | NS |
| CC | 0.0034 | 0.0016 | 2.0533 | 1.00E+00 | 0.0091 | 0.0001 | 0.0360 | NS |
| SCC | 0.0009 | 0.0013 | 0.6331 | 1.00E+00 | 0.0009 | 0.0000 | 0.0159 | NS |
| *AverageFA* | 0.0017 | 0.0009 | 1.8859 | 1.00E+00 | 0.0073 | 0.0000 | 0.0325 | NS |

*p_corr_<0.05; **p_corr_<0.01; ***p_corr_<0.001; ns: not significant

**Table S16.** **Mega-analysis:** **Linear mixed model results for the main effect of history of psychotic symptoms after controlling for age and sex**

| **ROI** | ***β*** | ***s.e*** | ***t*-value** | **P_corr_>\|*t*\|** | **R^2^** | **[0.025** | **0.975]** | **Sign.** |
| --- | --- | --- | --- | --- | --- | --- | --- | --- |
| *Projection fibers* | |  |  |  |  |  |  |  |
| ACR.L | -0.0012 | 0.0022 | -0.5402 | 1.00E+00 | 0.0004 | 0.0000 | 0.0072 | NS |
| ACR.R | -0.0013 | 0.0024 | -0.5327 | 1.00E+00 | 0.0004 | 0.0000 | 0.0072 | NS |
| ALIC.L | -0.0003 | 0.0021 | -0.1477 | 1.00E+00 | 0.0000 | 0.0000 | 0.0057 | NS |
| ALIC.R | -0.0002 | 0.0022 | -0.1122 | 1.00E+00 | 0.0000 | 0.0000 | 0.0056 | NS |
| CR.L | -0.0008 | 0.0018 | -0.4293 | 1.00E+00 | 0.0002 | 0.0000 | 0.0067 | NS |
| CR.R | 0.0001 | 0.0018 | 0.0423 | 1.00E+00 | 0.0000 | 0.0000 | 0.0055 | NS |
| CST.L | -0.0038 | 0.0033 | -1.1308 | 1.00E+00 | 0.0017 | 0.0000 | 0.0112 | NS |
| CST.R | -0.0019 | 0.0032 | -0.5965 | 1.00E+00 | 0.0005 | 0.0000 | 0.0075 | NS |
| IC.L | -0.0006 | 0.0016 | -0.3550 | 1.00E+00 | 0.0002 | 0.0000 | 0.0063 | NS |
| IC.R | 0.0000 | 0.0017 | -0.0149 | 1.00E+00 | 0.0000 | 0.0000 | 0.0055 | NS |
| PCR.L | 0.0016 | 0.0021 | 0.7485 | 1.00E+00 | 0.0008 | 0.0000 | 0.0085 | NS |
| PCR.R | 0.0033 | 0.0021 | 1.5751 | 1.00E+00 | 0.0033 | 0.0000 | 0.0149 | NS |
| PLIC.L | -0.0024 | 0.0022 | -1.0963 | 1.00E+00 | 0.0016 | 0.0000 | 0.0110 | NS |
| PLIC.R | 0.0000 | 0.0022 | 0.0013 | 1.00E+00 | 0.0000 | 0.0000 | 0.0055 | NS |
| PTR.L | -0.0017 | 0.0027 | -0.6045 | 1.00E+00 | 0.0005 | 0.0000 | 0.0076 | NS |
| PTR.R | -0.0001 | 0.0027 | -0.0246 | 1.00E+00 | 0.0000 | 0.0000 | 0.0055 | NS |
| RLIC.L | -0.0011 | 0.0022 | -0.4927 | 1.00E+00 | 0.0003 | 0.0000 | 0.0070 | NS |
| RLIC.R | 0.0004 | 0.0024 | 0.1453 | 1.00E+00 | 0.0000 | 0.0000 | 0.0057 | NS |
| SCR.L | -0.0011 | 0.0021 | -0.5232 | 1.00E+00 | 0.0004 | 0.0000 | 0.0072 | NS |
| SCR.R | -0.0003 | 0.0020 | -0.1440 | 1.00E+00 | 0.0000 | 0.0000 | 0.0057 | NS |
| *Association fibers* | |  |  |  |  |  |  |  |
| CGC.L | -0.0035 | 0.0032 | -1.0975 | 1.00E+00 | 0.0016 | 0.0000 | 0.0110 | NS |
| CGC.R | -0.0048 | 0.0031 | -1.5234 | 1.00E+00 | 0.0031 | 0.0000 | 0.0144 | NS |
| CGH.L | -0.0016 | 0.0035 | -0.4633 | 1.00E+00 | 0.0003 | 0.0000 | 0.0068 | NS |
| CGH.R | 0.0033 | 0.0037 | 0.8846 | 1.00E+00 | 0.0010 | 0.0000 | 0.0094 | NS |
| EC.L | -0.0014 | 0.0019 | -0.7280 | 1.00E+00 | 0.0007 | 0.0000 | 0.0084 | NS |
| EC.R | -0.0001 | 0.0019 | -0.0487 | 1.00E+00 | 0.0000 | 0.0000 | 0.0055 | NS |
| FX.ST.L | -0.0064 | 0.0028 | -2.2698 | 1.00E+00 | 0.0069 | 0.0004 | 0.0217 | NS |
| FX.ST.R | -0.0026 | 0.0030 | -0.8820 | 1.00E+00 | 0.0010 | 0.0000 | 0.0094 | NS |
| IFO.L | -0.0022 | 0.0035 | -0.6273 | 1.00E+00 | 0.0005 | 0.0000 | 0.0078 | NS |
| IFO.R | 0.0008 | 0.0033 | 0.2582 | 1.00E+00 | 0.0001 | 0.0000 | 0.0060 | NS |
| SFO.L | -0.0032 | 0.0028 | -1.1372 | 1.00E+00 | 0.0017 | 0.0000 | 0.0113 | NS |
| SFO.R | -0.0042 | 0.0027 | -1.5727 | 1.00E+00 | 0.0033 | 0.0000 | 0.0149 | NS |
| SS.L | -0.0028 | 0.0024 | -1.1941 | 1.00E+00 | 0.0019 | 0.0000 | 0.0118 | NS |
| SS.R | -0.0022 | 0.0025 | -0.8900 | 1.00E+00 | 0.0011 | 0.0000 | 0.0095 | NS |
| UNC.L | 0.0020 | 0.0040 | 0.5047 | 1.00E+00 | 0.0003 | 0.0000 | 0.0071 | NS |
| UNC.R | 0.0065 | 0.0038 | 1.7152 | 1.00E+00 | 0.0040 | 0.0000 | 0.0162 | NS |
| *Commissural fibers* | |  |  |  |  |  |  |  |
| BCC | -0.0034 | 0.0031 | -1.1091 | 1.00E+00 | 0.0017 | 0.0000 | 0.0111 | NS |
| CC | -0.0029 | 0.0023 | -1.2899 | 1.00E+00 | 0.0022 | 0.0000 | 0.0124 | NS |
| FX | -0.0027 | 0.0060 | -0.4591 | 1.00E+00 | 0.0003 | 0.0000 | 0.0069 | NS |
| GCC | -0.0021 | 0.0026 | -0.8192 | 1.00E+00 | 0.0009 | 0.0000 | 0.0089 | NS |
| SCC | -0.0025 | 0.0020 | -1.2569 | 1.00E+00 | 0.0021 | 0.0000 | 0.0121 | NS |
| *AverageFA* | -0.0003 | 0.0013 | -0.2259 | 1.00E+00 | 0.0001 | 0.0000 | 0.0058 | NS |

*p_corr_<0.05; **p_corr_<0.01; ***p_corr_<0.001; ns: not significant

**Table S17.** **Mega-analysis:** **Linear mixed model results for the main effect of type of bipolar disorder after controlling for age and sex**

| **ROI** | ***β*** | ***s.e*** | ***t*-value** | **P_corr_>\|*t*\|** | **R^2^** | **[0.025** | **0.975]** | **Sign.** |
| --- | --- | --- | --- | --- | --- | --- | --- | --- |
| *Projection fibers* | |  |  |  |  |  |  |  |
| PLIC.R | -0.0085 | 0.0038 | -2.1963 | 1.00E+00 | 0.0138 | 0.0013 | 0.0392 | NS |
| PLIC.L | -0.0081 | 0.0041 | -2.0027 | 1.00E+00 | 0.0114 | 0.0006 | 0.0353 | NS |
| IC.R | -0.0049 | 0.0029 | -1.7031 | 1.00E+00 | 0.0082 | 0.0002 | 0.0296 | NS |
| IC.L | -0.0043 | 0.0028 | -1.5501 | 1.00E+00 | 0.0068 | 0.0001 | 0.0269 | NS |
| ALIC.L | -0.0055 | 0.0036 | -1.5176 | 1.00E+00 | 0.0066 | 0.0001 | 0.0265 | NS |
| ALIC.R | -0.0051 | 0.0039 | -1.3134 | 1.00E+00 | 0.0050 | 0.0000 | 0.0232 | NS |
| SCR.R | -0.0035 | 0.0034 | -1.0117 | 1.00E+00 | 0.0030 | 0.0000 | 0.0186 | NS |
| SCR.L | -0.0036 | 0.0036 | -0.9998 | 1.00E+00 | 0.0029 | 0.0000 | 0.0185 | NS |
| CR.L | -0.0023 | 0.0031 | -0.7515 | 1.00E+00 | 0.0016 | 0.0000 | 0.0151 | NS |
| ACR.L | -0.0020 | 0.0038 | -0.5227 | 1.00E+00 | 0.0008 | 0.0000 | 0.0123 | NS |
| CR.R | -0.0016 | 0.0032 | -0.5032 | 1.00E+00 | 0.0007 | 0.0000 | 0.0121 | NS |
| PTR.L | 0.0020 | 0.0045 | 0.4418 | 1.00E+00 | 0.0006 | 0.0000 | 0.0115 | NS |
| RLIC.L | 0.0014 | 0.0040 | 0.3627 | 1.00E+00 | 0.0004 | 0.0000 | 0.0107 | NS |
| CST.L | -0.0019 | 0.0062 | -0.2980 | 1.00E+00 | 0.0003 | 0.0000 | 0.0101 | NS |
| PCR.R | -0.0010 | 0.0037 | -0.2698 | 1.00E+00 | 0.0002 | 0.0000 | 0.0099 | NS |
| CST.R | -0.0009 | 0.0059 | -0.1509 | 1.00E+00 | 0.0001 | 0.0000 | 0.0093 | NS |
| PCR.L | -0.0006 | 0.0037 | -0.1620 | 1.00E+00 | 0.0001 | 0.0000 | 0.0093 | NS |
| ACR.R | -0.0004 | 0.0042 | -0.0863 | 1.00E+00 | 0.0000 | 0.0000 | 0.0090 | NS |
| PTR.R | -0.0006 | 0.0045 | -0.1286 | 1.00E+00 | 0.0000 | 0.0000 | 0.0092 | NS |
| RLIC.R | -0.0002 | 0.0042 | -0.0446 | 1.00E+00 | 0.0000 | 0.0000 | 0.0090 | NS |
| *Association fibers* | |  |  |  |  |  |  |  |
| CGC.R | 0.0064 | 0.0052 | 1.2306 | 1.00E+00 | 0.0043 | 0.0000 | 0.0218 | NS |
| SS.L | 0.0048 | 0.0042 | 1.1503 | 1.00E+00 | 0.0038 | 0.0000 | 0.0207 | NS |
| SFO.L | -0.0055 | 0.0051 | -1.0784 | 1.00E+00 | 0.0034 | 0.0000 | 0.0196 | NS |
| SS.R | 0.0046 | 0.0043 | 1.0671 | 1.00E+00 | 0.0033 | 0.0000 | 0.0194 | NS |
| IFO.L | 0.0046 | 0.0062 | 0.7354 | 1.00E+00 | 0.0016 | 0.0000 | 0.0149 | NS |
| IFO.R | -0.0036 | 0.0060 | -0.6039 | 1.00E+00 | 0.0011 | 0.0000 | 0.0133 | NS |
| UNC.L | -0.0028 | 0.0068 | -0.4176 | 1.00E+00 | 0.0005 | 0.0000 | 0.0112 | NS |
| CGC.L | 0.0020 | 0.0053 | 0.3799 | 1.00E+00 | 0.0004 | 0.0000 | 0.0108 | NS |
| CGH.L | -0.0026 | 0.0067 | -0.3912 | 1.00E+00 | 0.0004 | 0.0000 | 0.0109 | NS |
| FX.ST.R | 0.0019 | 0.0051 | 0.3702 | 1.00E+00 | 0.0004 | 0.0000 | 0.0108 | NS |
| CGH.R | -0.0024 | 0.0070 | -0.3496 | 1.00E+00 | 0.0003 | 0.0000 | 0.0105 | NS |
| EC.L | -0.0010 | 0.0033 | -0.3109 | 1.00E+00 | 0.0003 | 0.0000 | 0.0103 | NS |
| FX.ST.L | -0.0014 | 0.0048 | -0.2943 | 1.00E+00 | 0.0002 | 0.0000 | 0.0101 | NS |
| EC.R | -0.0007 | 0.0034 | -0.1985 | 1.00E+00 | 0.0001 | 0.0000 | 0.0095 | NS |
| SFO.R | 0.0002 | 0.0049 | 0.0322 | 1.00E+00 | 0.0000 | 0.0000 | 0.0090 | NS |
| UNC.R | -0.0005 | 0.0067 | -0.0750 | 1.00E+00 | 0.0000 | 0.0000 | 0.0090 | NS |
| *Commissural fibers* | |  |  |  |  |  |  |  |
| BCC | 0.0077 | 0.0053 | 1.4441 | 1.00E+00 | 0.0060 | 0.0000 | 0.0253 | NS |
| FX | 0.0123 | 0.0098 | 1.2608 | 1.00E+00 | 0.0046 | 0.0000 | 0.0224 | NS |
| CC | 0.0043 | 0.0039 | 1.1003 | 1.00E+00 | 0.0034 | 0.0000 | 0.0198 | NS |
| SCC | 0.0032 | 0.0036 | 0.8976 | 1.00E+00 | 0.0023 | 0.0000 | 0.0169 | NS |
| GCC | -0.0001 | 0.0045 | -0.0229 | 1.00E+00 | 0.0000 | 0.0000 | 0.0089 | NS |
| *AverageFA* | -0.0001 | 0.0023 | -0.0619 | 1.00E+00 | 0.0000 | 0.0000 | 0.0090 | NS |

*p_corr_<0.05; **p_corr_<0.01; ***p_corr_<0.001; ns: not significant

**Table S18. Meta-analysis on bilateral tracts:** Cohen’s *d* values, their *s.e*., P-values and I^2^ values (heterogeneity between sites) sorted by effect size (descending order) for FA differences between patients with bipolar disorder and healthy controls after controlling for age and sex.

| **ROI** | **Cohen’s *d*** | ***s.e.*** | ***p*-value** | **I^2^** | ***p*-value (corr)** | **Sign.** |
| --- | --- | --- | --- | --- | --- | --- |
| *Projection fibers* | |  |  |  |  |  |
| PTR | -0.3028 | 0.0435 | 3.42E-12 | 12.5713 | 2.67E-08 | *** |
| ACR | -0.2431 | 0.0500 | 1.15E-06 | 30.8127 | 3.74E-05 | *** |
| CR | -0.1969 | 0.0552 | 3.63E-04 | 42.6360 | 8.83E-03 | ** |
| ALIC | -0.1532 | 0.0527 | 3.64E-03 | 37.3747 | 2.06E-01 | NS |
| PCR | -0.1524 | 0.0504 | 2.50E-03 | 32.0257 | 2.20E-01 | NS |
| SCR | -0.0860 | 0.0574 | 1.34E-01 | 46.9618 | 1.00E+00 | NS |
| IC | -0.0665 | 0.0555 | 2.31E-01 | 43.3278 | 1.00E+00 | NS |
| RLIC | -0.0471 | 0.0563 | 4.03E-01 | 44.8564 | 1.00E+00 | NS |
| CST | 0.0002 | 0.0656 | 9.98E-01 | 59.2586 | 1.00E+00 | NS |
| PLIC | 0.0363 | 0.0495 | 4.64E-01 | 29.9668 | 1.00E+00 | NS |
| *Association fibers* | |  |  |  |  |  |
| CGC | -0.3892 | 0.0594 | 5.83E-11 | 49.4365 | 4.58E-08 | *** |
| UNC | -0.2479 | 0.0519 | 1.80E-06 | 35.3111 | 2.26E-04 | *** |
| EC | -0.2298 | 0.0453 | 3.95E-07 | 18.3200 | 3.66E-05 | *** |
| SLF | -0.2266 | 0.0496 | 4.92E-06 | 29.9764 | 3.66E-04 | *** |
| SS | -0.1954 | 0.0494 | 7.71E-05 | 29.6040 | 4.33E-03 | ** |
| IFO | -0.1885 | 0.0393 | 1.60E-06 | 0.0000 | 9.45E-05 | *** |
| FXST | -0.1560 | 0.0393 | 7.12E-05 | 0.0185 | 1.47E-02 | * |
| SFO | -0.1548 | 0.0535 | 3.84E-03 | 39.1925 | 1.57E-01 | NS |
| CGH | -0.0678 | 0.0454 | 1.35E-01 | 18.7281 | 1.00E+00 | NS |
| *Commissural fibers* | |  |  |  |  |  |
| CC | -0.4625 | 0.0552 | 5.08E-17 | 41.3049 | 7.86E-12 | *** |
| BCC | -0.4297 | 0.0524 | 2.32E-16 | 35.4795 | 5.41E-11 | *** |
| GCC | -0.3729 | 0.0662 | 1.78E-08 | 59.3947 | 6.87E-06 | *** |
| SCC | -0.3387 | 0.0532 | 1.97E-10 | 37.9064 | 5.66E-08 | *** |
| FX | -0.2876 | 0.0536 | 8.19E-08 | 39.0287 | 7.84E-05 | *** |
| *AverageFA* | -0.2604 | 0.0756 | 5.69E-04 | 69.2396 | 1.66E-01 | NS |

*p_corr_<0.05; **p_corr_<0.01; ***p_corr_<0.001; ns: not significant
